# Identification and Surveys of Promoting Plant Growth VOCs from Biocontrol Bacteria Paenibacillus peoriae GXUN15128

**DOI:** 10.1128/spectrum.04346-22

**Published:** 2023-03-29

**Authors:** Kun Wang, Ziyan Lin, Jin Dou, Mingguo Jiang, Naikun Shen, Jing Feng

**Affiliations:** a Guangxi Key Laboratory for Polysaccharide Materials and Modifications, School of Marine Sciences and Biotechnology, Guangxi Minzu University, Nanning, China

**Keywords:** *Arabidopsis thaliana*, *Paenibacillus peoriae*, plant growth promoting, transcriptome, rhizosphere, volatiles, component identification

## Abstract

The role of microbial volatile organic compounds (MVOCs) in promoting plant growth has received much attention. We isolated Paenibacillus peoriae from mangrove rhizosphere soil, which can produce VOCs to promote the growth of Arabidopsis thaliana seedlings, increase the aboveground biomass of *A. thaliana*, and increase the number of lateral roots of *A. thaliana*. The effects of different inoculation amounts and different media on the composition of MVOCs were studied by solid-phase microextraction/gas chromatography-mass spectrometry (SPME/GC-MS) and headspace sampler/GC-MS. We found that the growth medium influences the function and composition of MVOCs. To survey the growth-promoting functions, the transcriptome of the receptor *A. thaliana* was then determined. We also verified the inhibitory effect of the soluble compounds produced by *P. peoriae* on the growth of 10 pathogenic fungi. The ability of *P. peoriae* to produce volatile and soluble compounds to promote plant growth and disease resistance has shown great potential for application in the sustainability of agricultural production.

**IMPORTANCE** Microbial volatile organic compounds (MVOCs) have great potential as “gas fertilizers” for agricultural applications, and it is a promising research direction for the utilization of microbial resources. This study is part of the field of interactions between microorganisms and plants. To study the function and application of microorganisms from the perspective of VOCs is helpful to break the bottleneck of traditional microbial application. At present, the study of MVOCs is lacking; there is a lack of functional strains, especially with plant-protective functions and nonpathogenic application value. The significance of this study is that it provides Paenibacillus peoriae, which produces VOCs with plant growth-promoting effects and broad-spectrum antifungal activity against plant-pathogenic fungi. Our study provides a more comprehensive, new VOC component analysis method and explains how MVOCs promote plant growth through transcriptome analysis. This will greatly increase our understanding of MVOC applications as a model for other MVOC research.

## INTRODUCTION

The interaction between microorganism and plant has caused widespread interest. As a special biological resource, microorganisms provide infinite possibilities for the development of agriculture. Microbial volatile organic compounds (MVOCs), a new medium of interaction between microorganisms and plants, induce plant systemic resistance (1) and morphological changes (2) directly or indirectly in the form of signal molecules. The main functions of volatile secondary metabolites of microorganisms are inhibiting plant pathogens (3), promoting plant growth (4), inducing plant systemic resistance (5), and enhancing plant stress resistance (6). Volatile organic compounds have a variety of types and functions. Based on the classification of 1,093 MVOCs recorded in the MVOCs database, the main microbial volatiles were terpenoids, alcohols, ketones, and aromatic compounds and organic acids, and each type accounts for more than 10% of the total volatile substances in the database, accounting for 64% of the total diversity (7). Aldehydes, sulfur and nitrogen compounds, alkanes, olefins, furans, esters, and ethers accounted for 37% of the total diversity. Among them, the most studied MVOCs with growth-promoting functions are 1-hexanol, indole, pentadecane, 3-hydroxy-2-butanone, 3-hexanone, acetophenone, and dimethyl disulfide. In addition to promoting growth, MVOCs are also the medium of bacteria-bacteria interactions and bacteria-fungi interactions (7, 8).

MVOCs can be used as “gaseous fertilizers” because of their plant growth-promoting properties (9), as a new type of biological fertilizer with broad prospects in agricultural production. The growth-promoting function of bacterial VOCs was first discovered by Ryu (10), then the growth-promoting function of fungal VOCs was also reported and began to be studied (11, 12). Fungal volatile organic compounds have attracted much attention because of their wide distribution and diverse composition. *Trichoderma* spp. and *Cladosporium* sp. are the most reported VOC-producing plant growth-promoting fungi (13, 14). *Trichoderma* spp. have been reported many times for their plant growth-promoting and antipathogenic functions (15–18). The functions of VOCs in bacteria, which have the largest numbers and the most species, have gained increased attention. *Bacillus*, Serratia, and Pseudomonas are the most studied genera of MVOCs, among which *Bacillus* is widely studied for its strong vitality and proliferation. VOCs from specific *Bacillus* species, such as Bacillus amyloliquefaciens (19–21), Bacillus subtilis (22, 23), and Bacillus methylotrophicus (24), have been found to promote plant growth by increasing auxin activity and auxin-dependent lateral root development, and VOCs were identified. Additionally, VOCs produced by Pseudomonas putida (25), *Azospirillum brasiliensis* (26), *Herbaspirillum* (26), *Microbacterium* (27), Acinetobacter (28), Pseudomonas (29), and Alcaligenes faecalis (30) have also been reported to promote the growth of *A. thaliana* (25, 29), *Brassica napus* (26), perennial ryegrass (27), lettuce and tomato (28), *Capsicum annuum* (29), and Solanum tuberosum (30), respectively. The substances in the above strains that have been shown to promote plant growth include propionic acid, benzene propanoic acid, butyric acid, 2,5-dimethylpyrazine, 3-methyl-1-butanol, benzyl alcohol, isoamyl propionate, 2,5-dimethylpyrazine, 2-heptanone, 2,3-butanedione, and 3-hydroxy-2-butanone. The discovery of 2,5-bimethylpyrazine made us realize the functional properties of pyrazine compounds. The functional VOCs of the above-mentioned strains were not duplicated, and the functional VOCs of different strains have certain specificity. However, other compounds with unverified function have reproducibility in VOCs from different strains, such as 1-butanol, 1-decanol, 1-octanol, 1-tridecanol, 2-decanone, 2-heptanone, 2-nonanol, 2-nonanone, 2-tridecanone, 2-undecanol, 2-undecanone, 3-methylbutanal, acetophenone, benzaldehyde, decanal, dodecane, nonanal, octanal, and pentanal. There are many kinds of microorganisms with VOCs that promote plant growth, and their functional components are different. Therefore, the discovery of new VOC functional strains and functional components is of great significance for the application of MVOC research.

Paenibacillus peoriae is a beneficial bacterium reported to have biocontrol effects (31). In research, microorganisms that can promote the health of plants in many aspects are considered beneficial bacteria, and beneficial bacteria have great application potential in plant protection (32). However, in the study of plant growth-promoting functions of VOCs, VOCs cannot be used as the main criterion to evaluate the beneficial bacteria or that the VOCs of plant pathogens can also promote plant growth (33). The production of VOCs by Pseudomonas aeruginosa Picf7 isolated from olive root can promote the growth of barley and Arabidopsis thaliana (34), VOCs produced by Rhizoctonia solani can promote the growth of *A. thaliana* seedlings and adult seedlings (35), VOCs produced by Botrytis cinerea QT5-19 could promote the growth of tomato (36), and VOCs produced by Alternaria alternata promote the growth and flowering of *A. thaliana* (37). Although pathogens can also have VOCs that have plant growth-promoting functions, studies of VOC growth-promoting functions based on beneficial bacteria have broader application prospects and have become the focus of MVOC research (38–44).

*P. peoriae* was originally named by the Heyndrickx specification (45). The study on the function of *P. peoriae* is limited to the fungistasis *in vitro*, which mainly focus on contact inhibition of the growth of pathogenic bacteria and antagonize the growth of Fusarium (46), Fusarium oxysporum (31), Staphylococcus aureus, Escherichia coli, and Candida albicans (47). Other effects besides bacteriostasis have not been reported. Studies of other *Paenibacillus* spp. have found that *Paenibacillus* promotes the growth of a variety of plants, including corn, poplar, pumpkin, rice, and switchgrass, and it also has significant effects on plant disease resistance and the production of polymyxins and fusaricidins (48). However, in terms of VOC function, only Paenibacillus polymyxa has been found to have bacteriostatic and nematicidal functions (49, 50), and other biostimulatory functions of *Paenibacillus* have not been reported in plant growth promotion studies.

In this study, we investigated the effects of total volatiles emitted by *P. peoriae* on plant growth and the antagonistic effects of *P. peoriae* on plant-pathogenic fungi *in vitro*. The effects of inoculation amount and medium on the growth-promoting activity of VOCs were studied by changing the microbial culture conditions. An innovative compositional analysis method (solid-phase microextraction/gas chromatography-mass spectrometry [SPME/GC-MS] combined with headspace sampler [HS]/GC-MS) was used to analyze the composition and content of VOCs. To determine the functional components in VOCs, individual compounds were used to verify the growth-promoting function. To further unravel the underlying molecular mechanisms of volatile-mediated growth promotion by *P. peoriae*, we conducted a genome-wide plant transcriptome analysis.

## RESULTS

### Screening of strains producing VOCs that promote plant growth.

Among 60 strains of soil rhizosphere bacteria isolated from mangrove rhizosphere soil, only 5 microbial strains had obvious growth-promoting effects through the observation of phenotype in coculture of microorganisms and plants. The growth-promoting effects of the above 5 microbial strains were evaluated by the “entropy weight method” (51); 1 strain with a strong plant growth-promoting function was screened and named GXUN15128 (Table S1 in the supplemental material). The VOC plant growth-promoting experiments of GXUN15128 were performed under different conditions. *A. thaliana* exposed to GXUN15128 volatiles in different media and with different inoculum amounts showed strong growth-promoting effects after 18 days. Plant growth parameters was calculated by measuring leaf length, leaf width, root length, and fresh weight of *A. thaliana* seedlings. Compared with the control group, the biomass of *A. thaliana* seedlings in the treatment group increased significantly.

**FIG 1:**
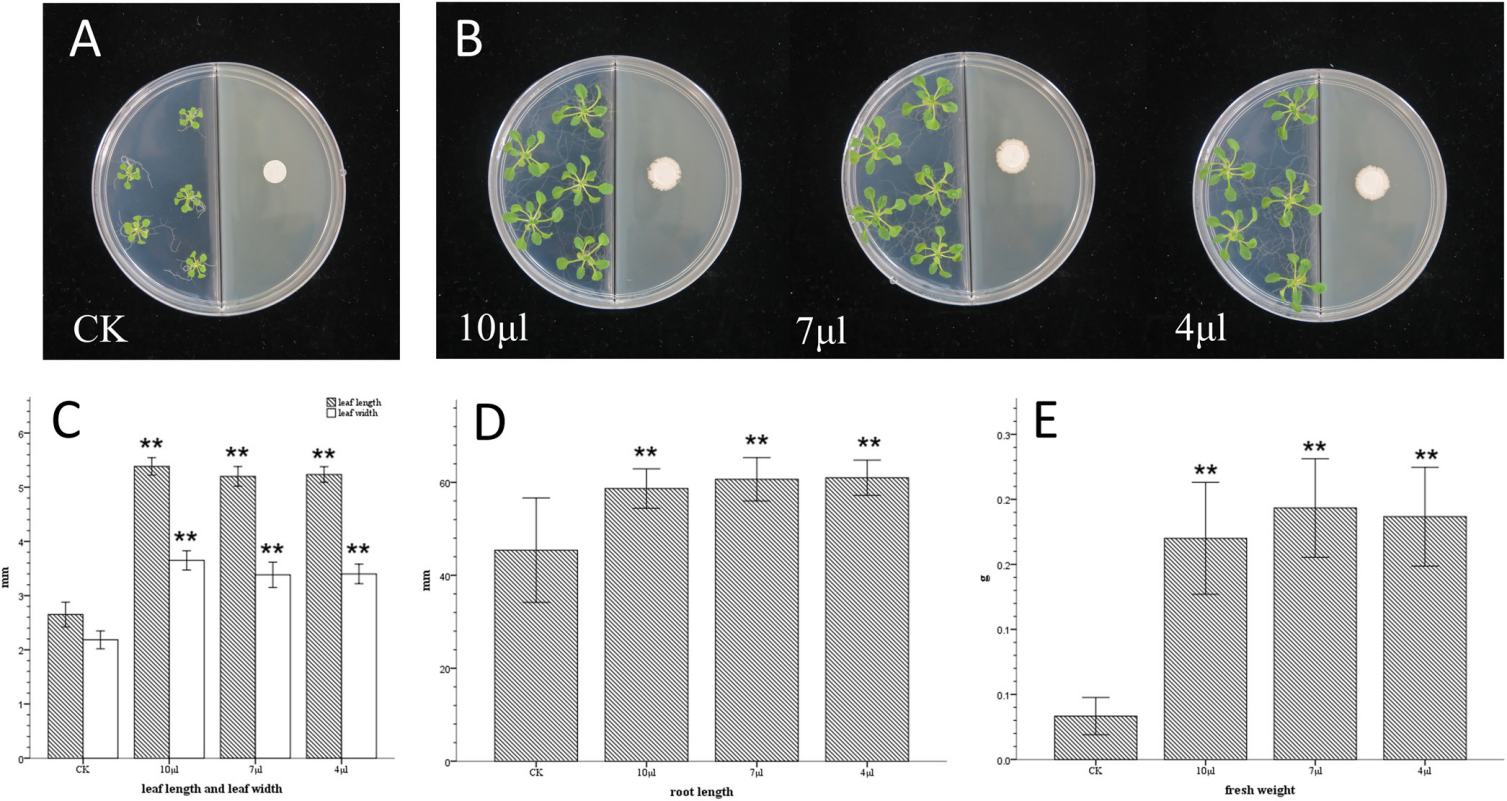
Effects of VOCs produced by different inoculation amounts of GXUN15128 cultured in LBA medium on the growth of Arabidopsis thaliana. (A) *A. thaliana* was cocultured on an I-plate and LBA medium with aseptic water and no physical contact. (B) *A. thaliana* was cocultured on an I-plate and LBA medium with GXUN15128 and no physical contact, and the inoculated volumes of 10^8^ CFU/mL suspension were 10 μL, 7 μL, and 4 μL (from left to right). (C) The effect of GXUN15128 on the length and width of leaves were as follows: CK was treated with aseptic water, and 10 μL, 7 μL, and 4 μL were 10^8^ CFU/mL GXUN15128; *, *P* < 0.05; **, *P* < 0.01. (D) Effects of GXUN15128 on root length. (E) Effects of GXUN15128 on fresh weight.

In Luria-Bertani agar (LBA) medium (Fig. 1), compared with the control group, the leaf lengths of the 10-μL, 7-μL, and 4-μL groups (10^8^ CFU/mL) increased by 103%, 96%, and 97%, respectively (*t* test, *P* < 0.01), the leaf widths increased by 67%, 54%, and 55%, respectively (*t* test, *P* < 0.01), and the fresh weights increased by 410%, 480%, and 460%, respectively (*t* test, *P* < 0.01).

**FIG 2:**
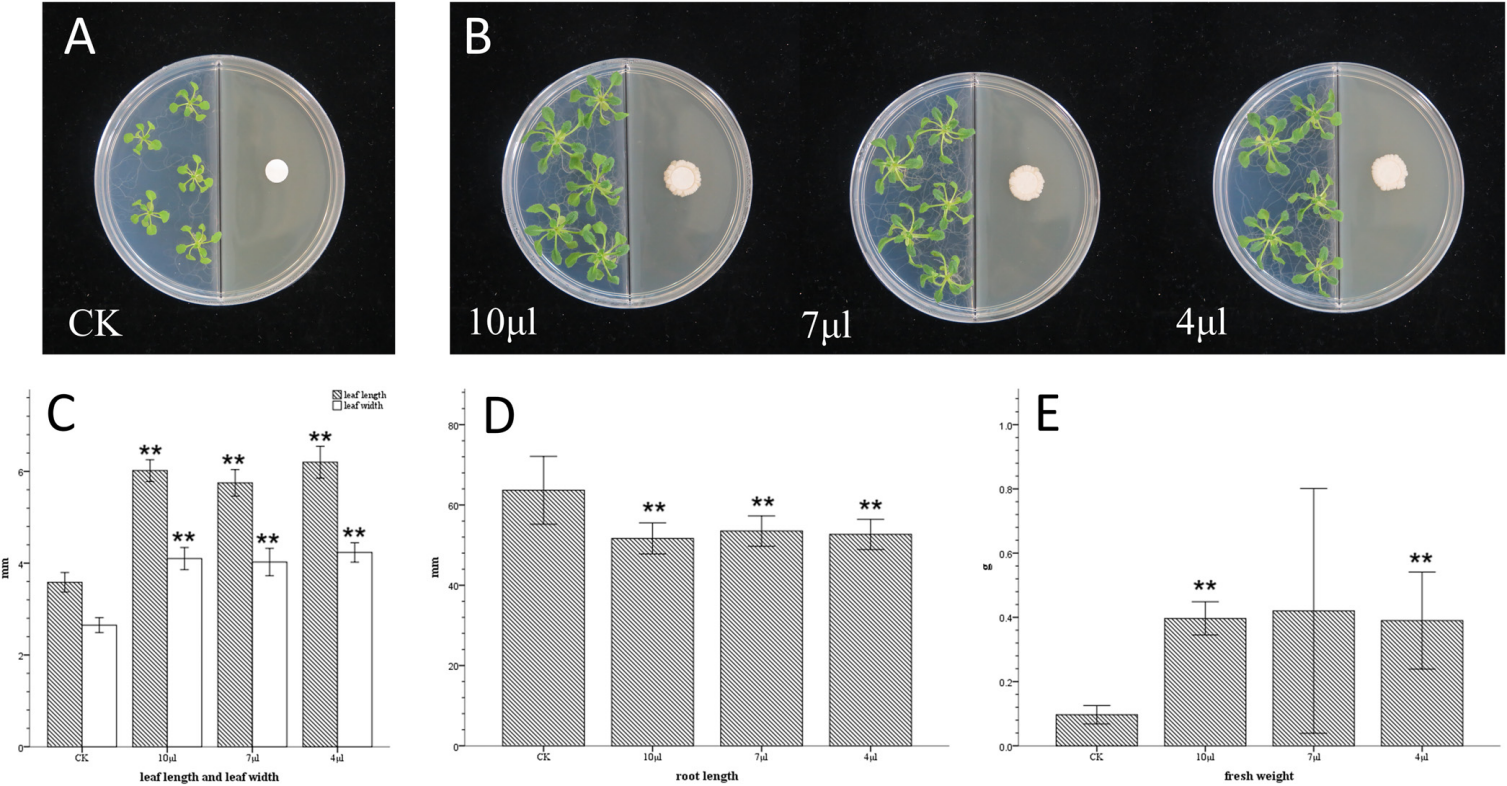
Effects of VOCs produced by different inoculation amounts of GXUN15128 cultured in TSA medium on the growth of Arabidopsis thaliana. (A) *A. thaliana* was cocultured on an I-plate and TSA medium with aseptic water and no physical contact. (B) *A. thaliana* was cocultured on an I-plate and TSA medium with GXUN15128 and no physical contact, and the inoculated volumes of 10^8^ CFU/mL suspension were 10 μL, 7 μL, and 4 μL (from left to right). (C) The effect of GXUN15128 on the length and width of leaves were as follows: CK was treated with aseptic water, 10 μL, 7 μL, and 4 μL were 10^8^ CFU/mL GXUN15128; *, *P* < 0.05; **, *P* < 0.01. (D) Effects of GXUN15128 on root length. (E) Effects of GXUN15128 on fresh weight.

In tryptose soya agar (TSA) medium (Fig. 2), compared with the control group, the leaf lengths of the 10-μL, 7-μL, and 4-μL groups increased by 68%, 60%, and 73%, respectively (*t* test, *P* < 0.01), the leaf widths increased by 54%, 52%, and 60%, respectively (*t* test, *P* < 0.01), and the fresh weights increased by 310%, 334%, and 303%, respectively (*t* test, *P* < 0.01).

The results showed that strain GXUN15128 had stable VOCs with plant-growth promoting function in LBA and TSA growth media and showed a certain amount of inoculum dependency, as larger amounts of inoculum induced larger transverse and longitudinal leaf diameters.

**FIG 3:**
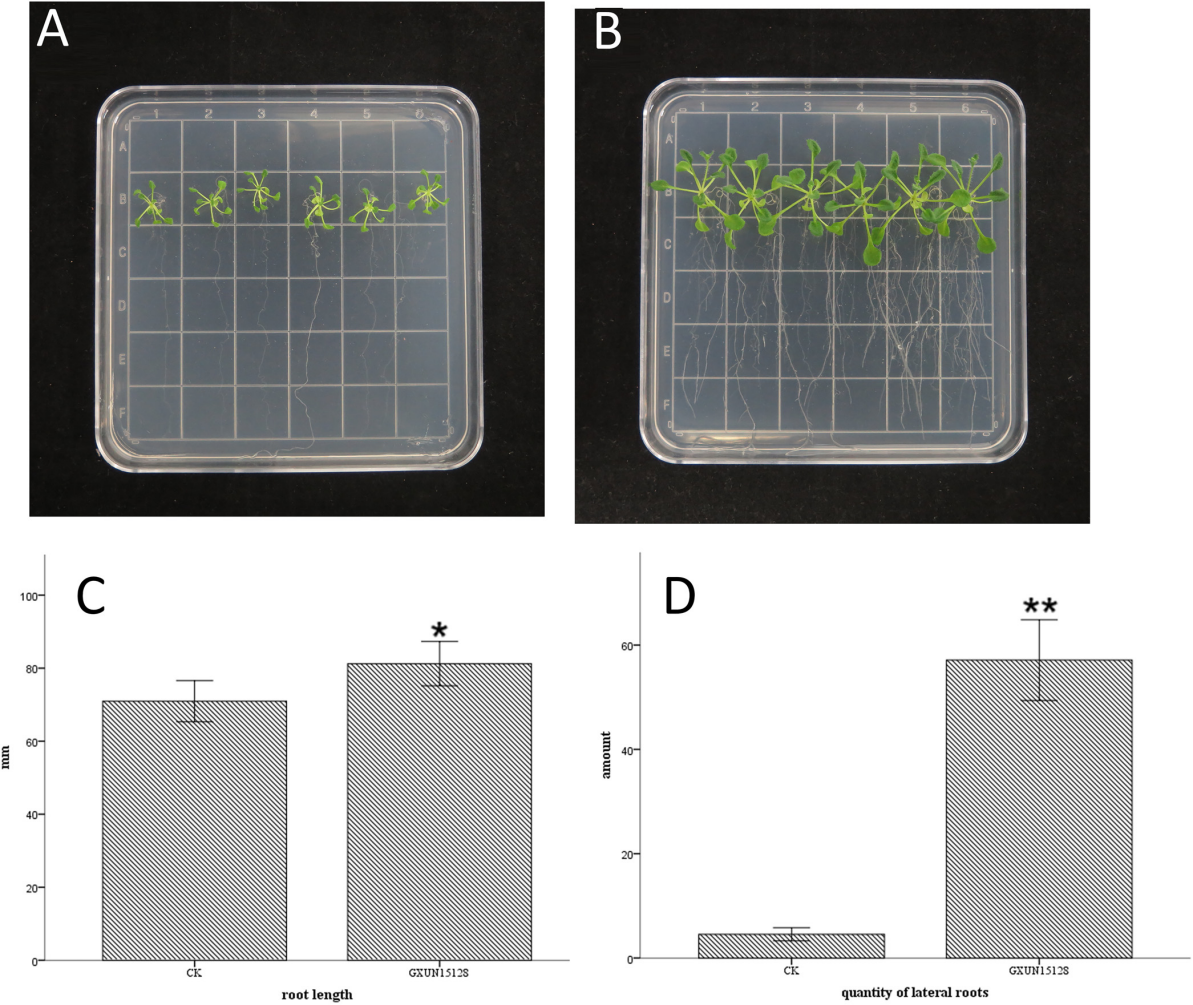
Effects of VOCs produced by GXUN15128 grown on TSA medium on root configuration of *A. thaliana.* (A) TSA medium was inoculated with sterile water and cocultured with *A. thaliana.* (B) TSA medium was inoculated with GXUN5128 and cocultured with *A. thaliana*. (C) Histogram of the effect of inoculation with sterile water and GXUN15128 on root length in *A. thaliana*; *, *P* < 0.05; **, *P* < 0.01. (D) Histogram of the effect of inoculation with sterile water and GXUN15128 on the number of lateral roots in *A. thaliana*.

VOCs of GXUN15128 had no significant effect on the root length of *A. thaliana*, but a significant increase in the number of lateral roots was observed. Therefore, we focused on the root effects on *A. thaliana* by using the double plate method and vertical culture. Two 10 cm × 10 cm × 2 cm square plates were used for the double plate method; one plate was filled with TSA medium, and the other plate was filled with Murashige and Skoog (MS) medium. Six *A. thaliana* seedlings were transplanted horizontally in MS plates at a distance of 3 cm from the plate boundary. TSA plates were inoculated with 10 μL of 10^8^ CFU/mL bacterial suspension on two paper discs. The results showed that the GXUN15128 VOC had no significant effect on the root length of *A. thaliana* seedlings, but the number of lateral roots increased by 1,154% (Fig. 3).

### Identification of Paenibacillus peoriae GXUN15128.

**FIG 4:**
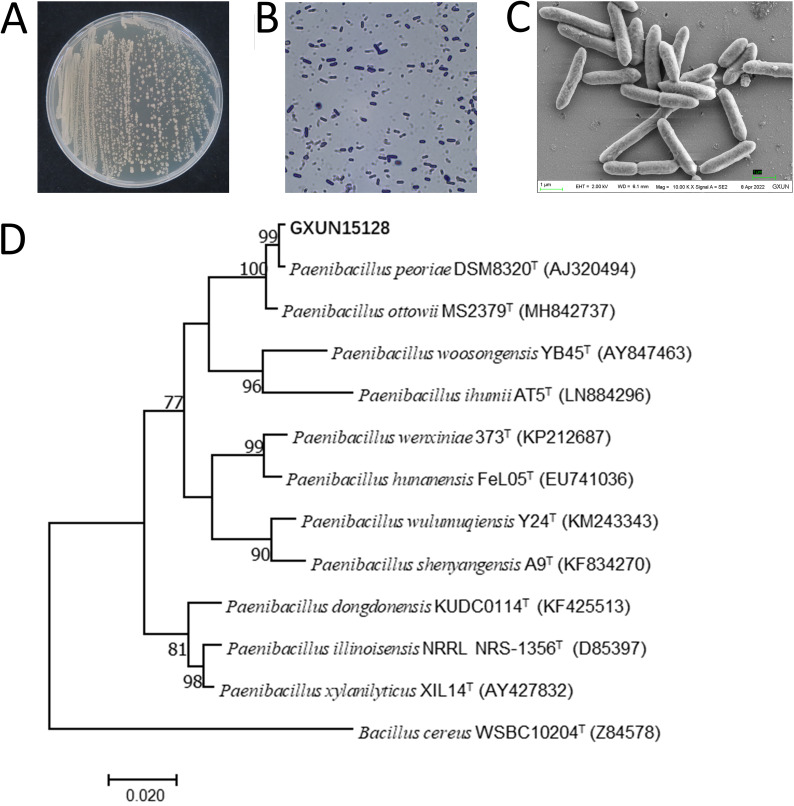
Morphological observation and phylogenetic tree of GXUN15128. (A) GXUN15128 colony morphology. (B) GXUN15128 Gram staining by light microscopy (×1,000); red is Gram negative, and purple is Gram positive. (C) GXUN15128 colony morphology by electron microscopy (×10,000). (D) The neighbor-joining tree based on the 16S rRNA gene sequences showing the phylogenetic relationship of GXUN15128 with related taxa. The evolutionary distances were computed using the p-distance method and are in the units of the number of base differences per site. The sequence of Bacillus cereus WSBC10204 (Z84578) was used as an outgroup. Bootstrap support values were calculated from 1,000 replicates, and only values above 60% are shown; scale bar = 0.005 substitutions per nucleotide position. Evolutionary analyses were conducted in MEGA7.

The bacterial strain GXUN15128 appeared whitish in color, had a shiny texture and sticky colonies, and emanated a strong smell. Gram-staining results are shown in purple, so GXUN15128 is Gram positive. When viewed through an electron microscope, GXUN15128 is a short, rod-shaped bacterium. The 16S rRNA gene sequence of GXUN15128 was determined and used for a BLAST search of the GenBank database of the National Center for Biotechnology Information (NCBI). The BLAST search revealed that strain GXUN15128 exhibited the highest gene sequence similarity with the *P. peoriae*. The phylogenetic tree was constructed by MEGA7 with 11 strains most similar to GXUN15128. The results showed that GXUN15128 was the closest relative to Paenibacillus peoriae. The average nucleotide identity (ANI) and digital DNA-DNA hybridization (dDDH) values of eight strains of *P. peoriae* were higher than the taxonomic thresholds of 95% and 70%, respectively, which indicated that GXUN15128 was *P. peoriae* (Table S2). Colony characteristics, Gram staining, electron microscopy, and phylogenetic analysis showed that GXUN15128 was *P. peoriae*, which was consistent with the Gram-positive bacteria of DSM8320 (Fig. 4).

### Identification of GXUN15128 volatiles.

To identify the chemical composition of the VOCs of GXUN15128 that promote *A. thaliana* growth, we created a closed environment that mimics VOC release in a coculture environment; VOC emission from GXUN15128 was enriched in headspace vials.

Notably, the bacterial growth medium we used was a precultured solid bacterial plug, and the sample treatment methods previously used to identify microbial VOC components were mainly in the form of liquid fermentation (28, 29). This method can increase the total amount of bacteria and secondary metabolites in the fermentation broth; but due to the complexity of VOC components, water-soluble substances will cause component loss and cannot reflect the true content of components, and the stability of the experimental results is reduced. The comparison between solid and liquid media shows that solid medium can improve the stability and reproducibility of experimental results, which has been applied in other research (34, 52, 53).

**FIG 5:**
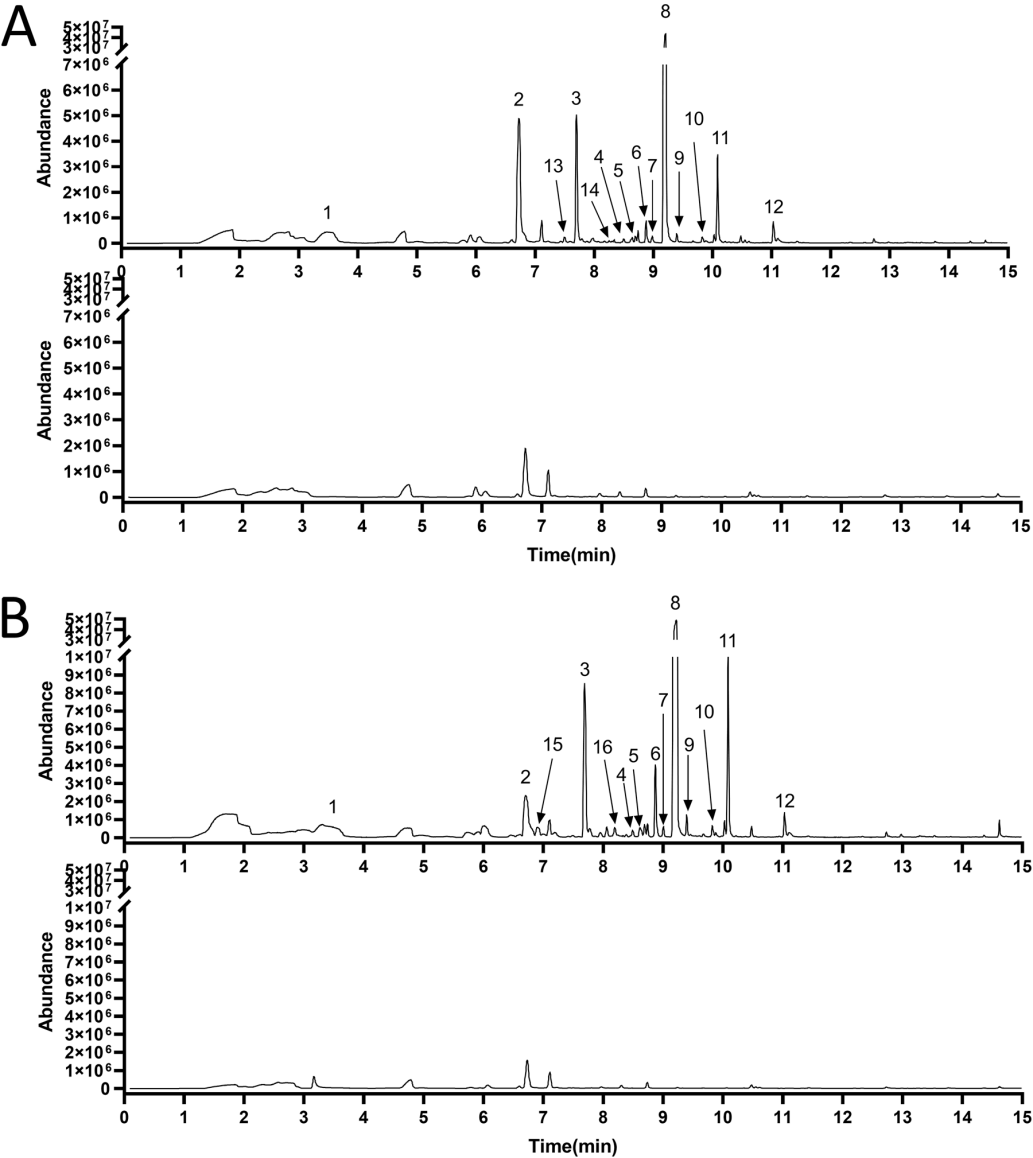
The meteorological chromatograms of GXUN15128 VOCs grown on LBA and TSA medium were analyzed by HS-SPME/GC-MS. (A) The gas components of GXUN15128 and LBA medium were detected by HS-SPME/GC-MS. The gas chromatograms obtained were compared, the different peaks were labeled, and the components were identified corresponding to Table 1. (B) The gas compositions of GXUN15128 and TSA were detected by HS-SPME/GC-MS. The gas chromatograms were compared, and the different peaks were labeled corresponding to Table 1.

We used composition analysis by the headspace-solid-phase microextraction/GC-MS (HS-SPME/GC-MS) method for sample pretreatment and composition analysis. Sixteen different compounds were identified among the VOCs from GXUN15128 (Fig. 5). Among them, 14 substances were identified in the LBA group, and 14 substances were identified in the TSA group. There were four different substances between the two experimental groups, 2-ethyl-1-hexanol, 2-nonanol, 1-octen-3-ol, and 2-methoxy-phenol. The changes of the other components were stable, and the identification results of 12 substances were consistent. The information on the identified ingredients is provided in Table 1.

**TABLE 1:** The VOC components of GXUN15128 grown on LB and TSA medium were analyzed by HS-SPME/GC-MS

Medium	Label	Metabolite	Retention time (min)	CAS no.	Sim score
LBA	1	1-Butanol, 2-methyl-	3.48	137-32-6	87.02
	2	Benzaldehyde	6.72	100-52-7	98.97
	3	2-Methyl-3-isopropylpyrazine	7.7	15986-81-9	93.77
	4	2,5-Dimethyl-3-isopropylpyrazine	8.5	13610-20-3	75.34
	5	2-Isobutyl-3-methylpyrazine	8.64	13925-06-9	86.43
	6	2,3,5-Trimethyl-6-propylpyrazine	8.88	92233-82-4	80.37
	7	1-Nonanol	8.98	143-08-8	91.26
	8	2,3,5-Trimethyl-6-propylpyrazine	9.21	92233-82-4	80.28
	9	2,3,5-Trimethyl-6-propylpyrazine	9.39	92233-82-4	82.19
	10	2-(2-Methylpropyl)-3-(1-methylethyl)pyrazine	9.83	1000108-60-3	84.13
	11	2-(2-Methylpropyl)-3-(1-methylethyl)pyrazine	10.09	1000108-60-3	91.94
	12	2-Dodecanone	11.03	6175-49-1	95.97
	13	1-Hexanol, 2-ethyl-	7.5	104-76-7	94.7
	14	2-Nonanol	8.25	628-99-9	82.98
TSA	1	1-Butanol, 2-methyl-	3.31	137-32-6	86.13
	2	Benzaldehyde	6.71	100-52-7	91.34
	3	2-Methyl-3-isopropylpyrazine	7.69	15986-81-9	93.83
	4	2,5-Dimethyl-3-isopropylpyrazine	8.49	13610-20-3	82.65
	5	2-Isobutyl-3-methylpyrazine	8.61	13925-06-9	87.41
	6	2,3,5-Trimethyl-6-propylpyrazine	8.87	92233-82-4	80.65
	7	1-Nonanol	9.01	143-08-8	94.12
	8	2,3,5-Trimethyl-6-propylpyrazine	9.22	92233-82-4	80.42
	9	2,3,5-Trimethyl-6-propylpyrazine	9.39	92233-82-4	82.2
	10	2-(2-Methylpropyl)-3-(1-methylethyl)pyrazine	9.82	1000108-60-3	88
	11	2-(2-Methylpropyl)-3-(1-methylethyl)pyrazine	10.09	1000108-60-3	91.38
	12	2-Dodecanone	11.03	6175-49-1	95.74
	15	1-Octen-3-ol	6.9	3391-86-4	94.67
	16	Phenol, 2-methoxy-	8.19	90-05-1	95.3

### Determination of the relative content of GXUN15128 volatiles.

To accurately analyze the relationship between the composition of microbial volatile substances, we controlled for two aspects of sample treatment and analysis methods. The sample preparation method adopts the solid culture medium method as described above. The headspace automatic sampler is used for sample collection and sampling. The principle is to automatically control the sampling temperature and conditions, control consistency and negative pressure to absorb the overhead VOCs, directly quantitative enrichment, and use the GC/MS system for analysis to collect and analyze. This method minimizes sample loss and accurately reflects the relationship between the content of each component.

The chromatographic peaks of the LBA and TSA groups were analyzed by GC-MS (Fig. S1), and the information on the identified ingredients is listed in Table S3. The main components of the LBA and TSA groups were the same, and the relationship between the main components was 2,3,5-trimethyl-6-propylpyrazin>benzaldehyde>2-(2-methylpropyl)-3-(1-methylethyl)pyrazine>2-methyl-3-isopropylpyrazine; other microconstituents were not detected. The results were consistent with those of HS-SPME/GC-MS.

### Growth-promoting activity of VOCs.

In addition to pyrazine compounds [2-methyl-3-isopropylpyrazine, 2,5-dimethyl-3-isopropylpyrazine, 2-isobutyl-3-methylpyrazine, 2,3,5-trimethyl-6-propylpyrazine, and 2-(2-methylpropyl)-3-(1-methylethyl)-pyrazine], which were not available for purchase, eight standard compounds (benzaldehyde, 1-nonanol, 2-dodecanone, 2-methyl-1-butanol, 2-ethyl-1-hexanol, 2-nonanol, 1-octen-3-ol, and 2-methoxy-phenol) were purchased for validation of plant growth-promoting activity based on GC-MS identification results. According to the growth parameters of *A. thaliana* (Fig. S2), the effects of different concentrations with the same compound on the growth of *A. thaliana* were similar in four aspects: leaf length, leaf width, root length, and fresh weight (Fig. S3). Among them, 2-ethyl-1-hexanol and 2-methoxy-phenol showed a weak effect of growth promotion but did not reach significance. Other compounds also had no significant growth-promoting effect.

### Transcriptomic response to VOC exposure.

**FIG 6:**
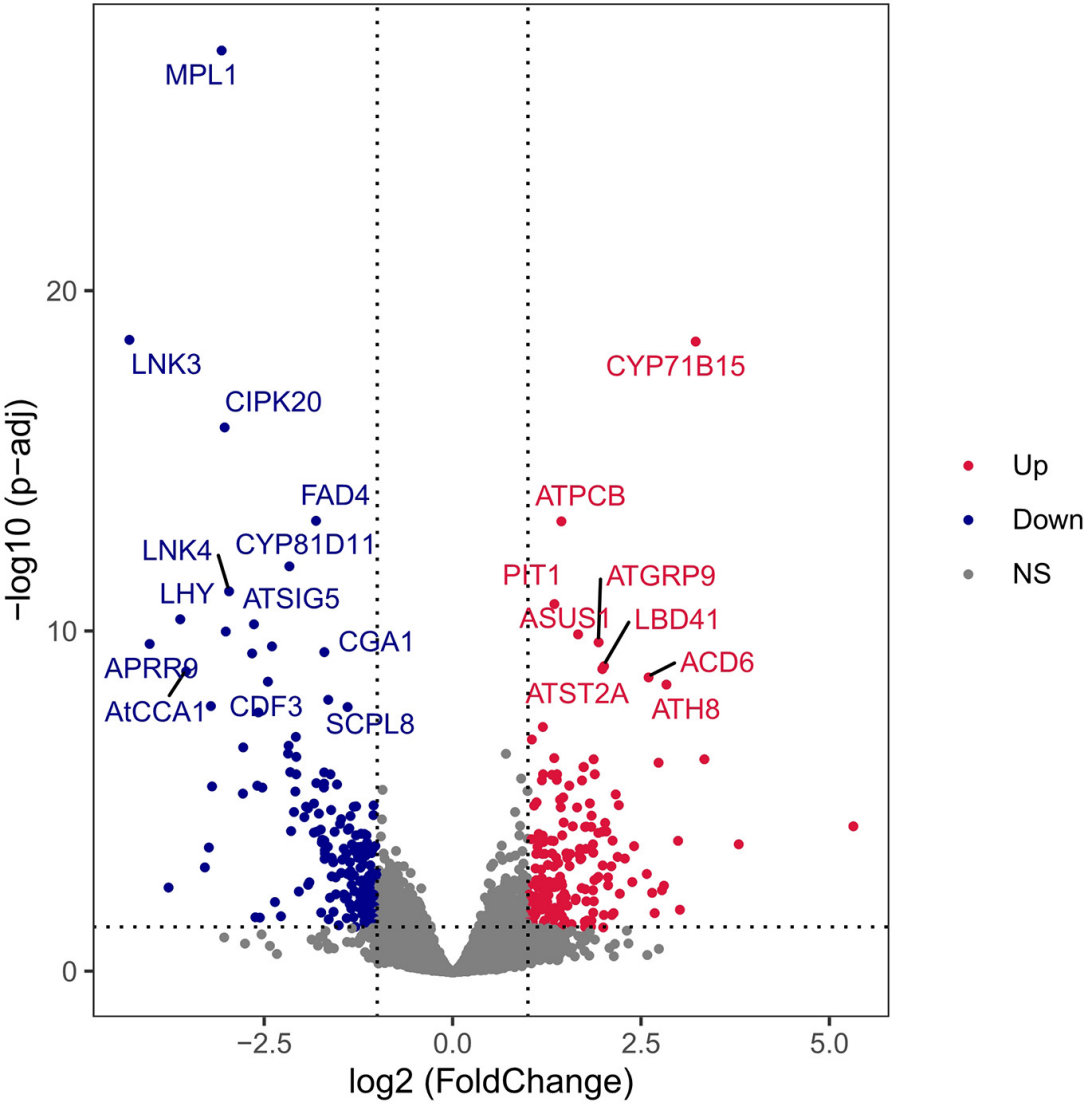
Differential gene volcano map. Differential gene volcano map showing the difference and reliability of differential gene expression between the treatment and control groups. Red represents upregulation of gene expression, and blue represents downregulation of gene expression; NS, not significant.

**FIG 7:**
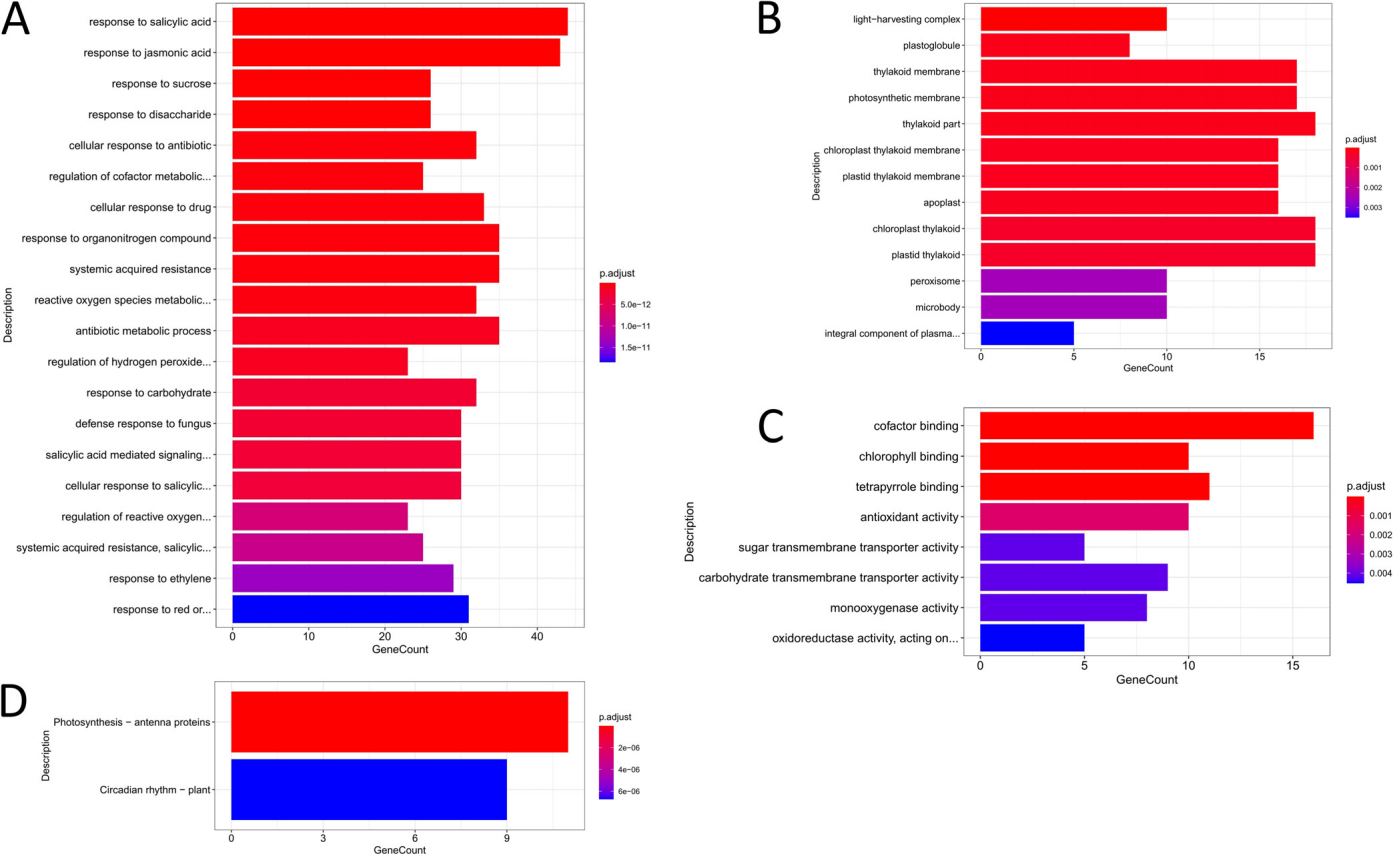
GO enrichment histogram of DEGS in BP, CC, and MF and KEGG enrichment histogram. (A) Enrichment results of BP in the enrichment of GO function. (B) Enrichment results of CC in the enrichment of GO function. (C) Enrichment results of MF in the enrichment of GO function. An adjusted *P* value of <0.05 was used as the threshold for GO functional enrichment. The bar chart shows the 20 most prominent GO pathways or all of them if fewer than 20. (D) Enrichment results of KEGG metabolic pathways. An adjusted *P* value of <0.05 was used as the threshold of significance.

Transcriptome data analysis showed that 677 differentially expressed genes (DEGs) were generated in the experimental group, including 365 DEGs with official gene symbol, and the top 20 genes in order of adjusted *P* value size were labeled in the differential gene volcano map (Fig. 6). Using adjusted *P* values of less than 0.05 as the threshold of significant enrichment, Gene Ontology (GO) function enrichment results were analyzed by biological process (BP), cellular component (CC), and molecular function (MF) (Fig. 7). The results showed that in BP, DEG functions were mainly concentrated in hormone regulation, redox metabolism, organic matter utilization, and far-red light reaction, while in CC, DEG functions were mainly concentrated in photosynthetic sites. In MF, DEG functions mainly focused on photosynthesis-related molecules. The DEGs were enriched in two KEGG metabolic pathways by KEGG enrichment analysis. Additional information is listed in Fig. S4 and Table S5.

**FIG 8:**
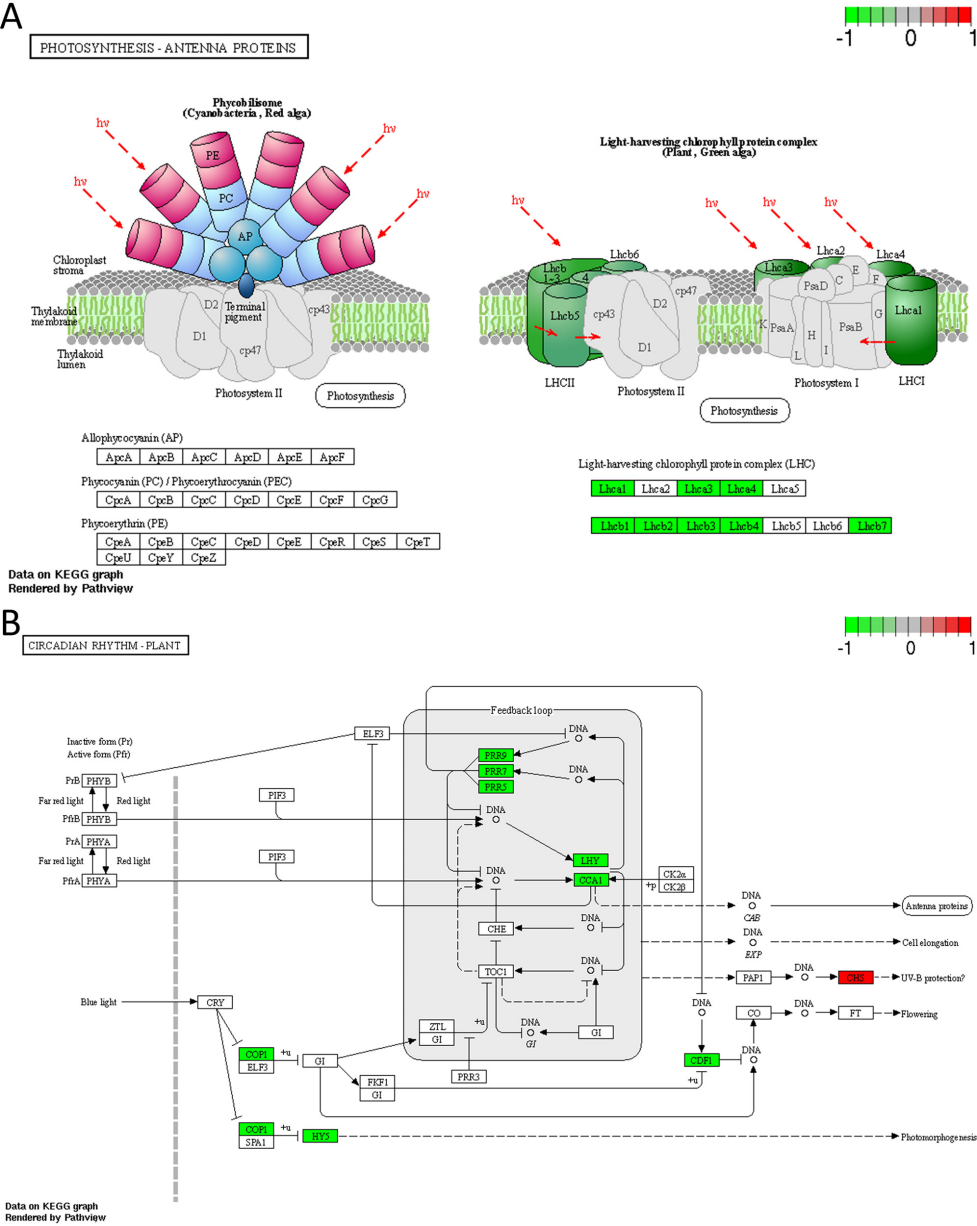
Photosynthesis antenna protein pathway and circadian rhythm plant pathway. (A) Photosynthesis antenna protein pathway. Red shows genes that are significantly upregulated, and green shows genes that are significantly downregulated. (B) Circadian rhythm plant pathway. Red shows genes that are significantly upregulated, and green shows genes that are significantly downregulated.

The two metabolic pathways were photosynthesis antenna proteins (KO 00196) in the metabolism classification and circadian rhythm plant (KO 04712) in the organismal systems classification. The chlorophyll-binding subunits of photosystem I (PSI) and PSII in the photosynthesis antenna protein pathway are the internal antenna light-trapping proteins for oxygen photosynthesis. In the photosynthesis antenna protein pathway (Fig. 8), the genes encoding light-harvesting complex I chlorophyll a/b binding protein 1/3/4 (*LHCA1*, *LHCA3*, and *LHCA4*) in PSI were downregulated; in PSII, the genes encoding light-harvesting complex II chlorophyll a/b binding protein 1/2/3/4/7 (*LHCB1*, *LHCB2*, *LHCB3*, *LHCB4*, and *LHCB7*) were also downregulated. Under the influence of VOCs produced by GXUN15128, *LHCA1*, *LHCA3*, and *LHCA4* were downregulated by 1.37-, 1.14-, and 1.14-fold, respectively.

From the circadian rhythm plant pathway (Fig. 8), the expression levels of *PRR5*/*PRR7*/*PRR9*, *LHY*, *CCA1*, constitutively photomorphogenic 1 (*COP1*), *HY5*, and *CDF1*, which encode pseudoresponse regulator (PRR), MYB-related transcription factor, circadian clock-associated 1, E3 ubiquitin-protein ligase RFWD2, transcription factor, and DOF zinc finger protein DOF5.5, respectively, were downregulated in plants. Only the gene encoding chalcone synthase (*CHS*) was upregulated and up to 1.28-fold. Expression of *LHY* and *CCA1* in the central oscillator decreased by 3.62- and 3.54-fold, respectively, and the expression of *PRR5*/*PRR7*/*PRR9* was also decreased by 1.36-, 1.54-, and 4.02-fold, respectively. The other circadian clock-related genes *COP1*, *HY5*, and *CDF1* decreased by 1.01-, 1.04-, and 1.37-fold, respectively.

### Quantitative real time polymerase chain reaction (qRT-PCR) verification of RNA-seq.

**FIG 9:**
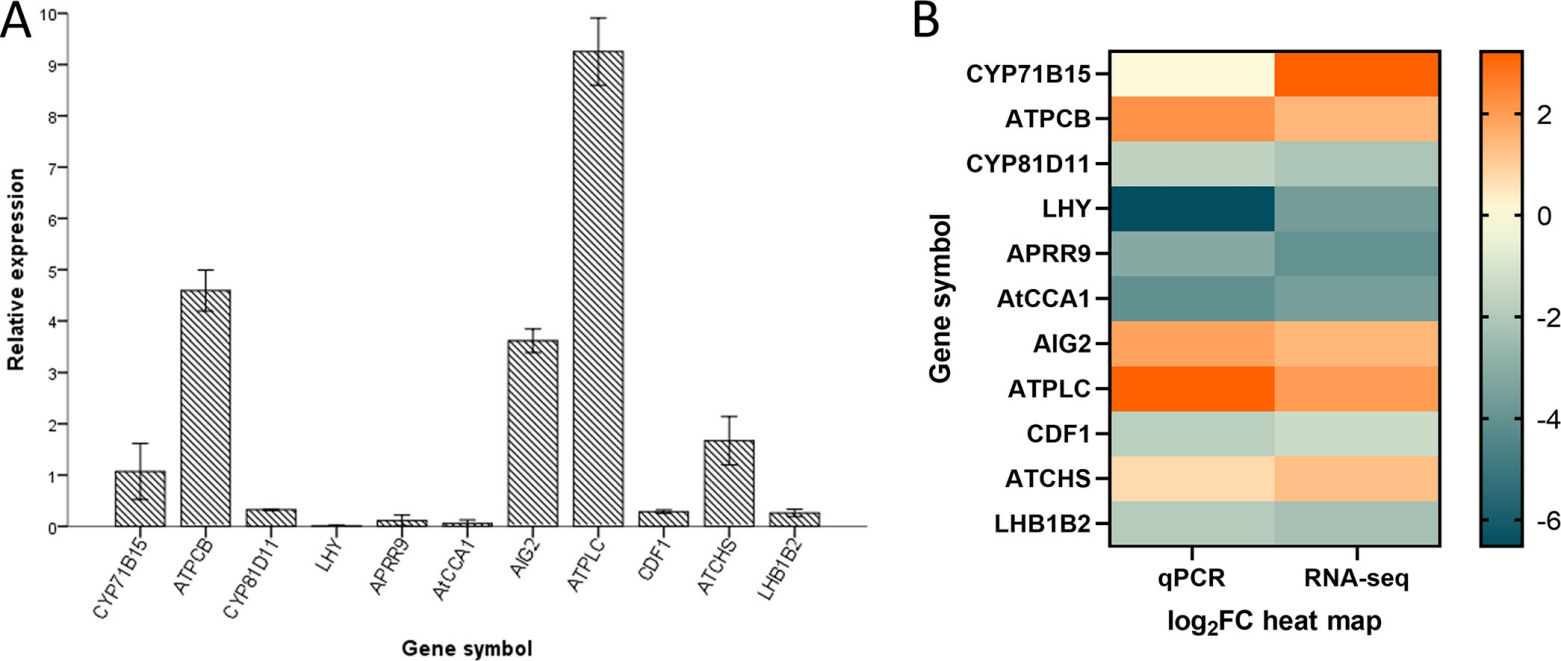
Results of quantitative PCR (qPCR) gene expression validation and heat map of comparison with transcriptome expression. (A) qRT-PCR was used to verify the expression levels of the major differentially expressed genes in the transcriptome of *A. thaliana* receptor in the experimental group; NM_113595, cytochrome P450 superfamily protein, *CYP71B15*; NM_114771, peroxidase CB, *ATPCB*; NM_113795, cytochrome P450 superfamily protein, *CYP81D11*; NM_099988, homeodomain-like superfamily protein, *LHY*; NM_130245, pseudoresponse regulator 9 (PRR9), *APRR9*; NM_180129, circadian clock-associated 1, *AtCCA1*; NM_113814, *AIG2*-like (avirulence-induced gene) family protein, *AIG2*; NM_125254, phospholipase C1, *ATPLC*; NM_125637, cycling DOF factor 1, *CDF1*; NM_121396, chalcone and stilbene synthase family protein, *ATCHS*; NM_128994, photosystem II light-harvesting complex protein B1B2, *LHB1B2*. The error bars represent standard error of four duplicate samples. The *y* axis reference line 1 represents the relative expression of the reference gene *Actin*. (B) After the relative expression level was treated with index 2 and the standard of transcriptome differential gene B was unified, the heat map was drawn.

According to GO functional annotation and KEGG metabolic pathway analysis, we selected 11 representative genes with significant differences for qRT-PCR validation (NM_113595, cytochrome P450 superfamily protein gene, *CYP71B15*; NM_114771, peroxidase CB, *ATPCB*; NM_113795, cytochrome P450 superfamily protein gene, *CYP81D11*; NM_099988, homeodomain-like superfamily protein gene, *LHY*; NM_130245, pseudoresponse regulator 9 (PRR9), *APRR9*; NM_180129, circadian clock-associated 1, *AtCCA1*; NM_113814, AIG2-like [avirulence induced gene] family protein gene, *AIG2*; NM_125254, phospholipase C1, *ATPLC*; NM_125637, cycling DOF factor 1, *CDF1*; NM_121396, chalcone and stilbene synthase family protein gene, *ATCHS*; NM_128994, photosystem II light-harvesting complex protein B1B2 gene, *LHB1B2*) (Table S4). We can see from the histogram of the relative expression and the heat map of the standardized treatment that the expression trend obtained in the qRT-PCR analysis was consistent with that in the transcriptome analysis (Fig. 9).

### Inhibitory effect of GXUN15128 on 10 plant pathogens *in vitro*.

**FIG 10:**
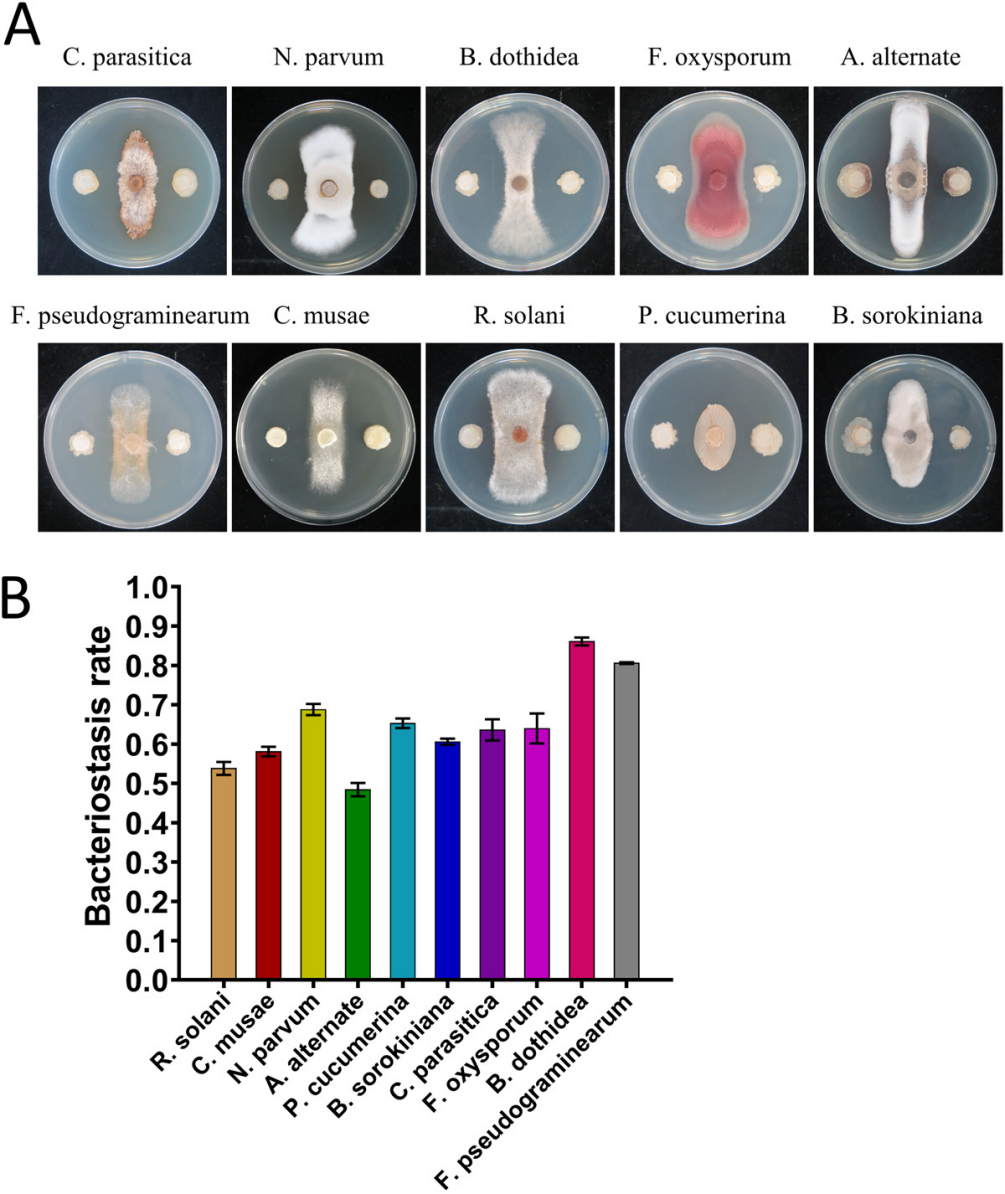
*In vitro* inhibitory effect of GXUN15128 on 10 plant-pathogenic fungi and its inhibitory rate. (A) GXUN15128 was inoculated in the left and right directions to represent the experimental group. The control group was treated with no bacteria in the upper and lower directions of the plate. (B) Column statistics table of the inhibitory effect and inhibition rate of GXUN15128 plate confrontation experiment on 10 plant-pathogenic fungi, including Rhizoctonia solani, *Colletotrichum musae*, *Neofusicoccum parvum*, *Alternaria alternate*, *Plectosphaerella cucumerina*, Bipolaris sorokiniana, *Cryphonectria parasitica*, Fusarium oxysporum, *Botryosphaeria dothidea*, and Fusarium *pseudograminearum*.

GXUN15128 showed strong inhibitory effects on 10 plant-pathogenic fungi by dual culture technique *in vitro* (Fig. 10). The results showed that GXUN15128 had the best inhibitory effect on *Botryosphaeria dothidea* and Fusarium *pseudograminearum*, reaching 86.1% and 80.6%, respectively, and had better inhibitory effects on *Neofusicoccum parvum*, *Plectosphaerella cucumerina*, Bipolaris sorokiniana, *Cryphonectria parasitica*, and Fusarium oxysporum, with bacteriostatic rates of 68.8, 48.4, 65.3, 60.6, 63.6, and 64.0%, respectively. GXUN15128 also had a strong inhibitory effect on *Alternaria alternate*, Rhizoctonia solani, and *Colletotrichum musae*, with bacteriostatic rates of 48.4, 53.8, and 58.1%, respectively. VOC antagonistic assays showed that VOCs produced by GXUN15128 had no significant inhibitory effect on 10 pathogenic fungi (Fig. S5).

## DISCUSSION

Studies have shown that microbial volatiles depend on culture conditions (54), including growth phase (21, 55), experimental model (56), oxygen content (57), growth medium (21, 29, 56, 58–60), temperature (61, 62), and pH (57, 63). Among them, the influence of growth medium is particularly important. Changes in specific components of the growth medium can cause changes in the volatile matter, with Staphylococcus aureus and Staphylococcus epidermidis producing different components in high-glucose and low-glucose media (59, 60). Shane also demonstrated the effect of glucose on the release of VOCs (58). Certain nutrients and amino acids also cause changes in terpenoid and sulfur-nitrogen compound metabolism (64), and we therefore used LBA and TSA to validate the VOC plant growth-promoting activity of GXUN15128 in different growth media. In addition, the microbial growth phase is an important factor affecting VOC release, and the number of bacteria has a significant impact on VOC release (21, 55). Therefore, we also set three volume gradients (10 μL, 7 μL, and 4 μL) of 10^8^ CFU/mL bacteria suspension. Although the growth-promoting effects of different inoculation doses had a tendency to increase with the increase of inoculation doses, there was no significant difference among them, and they were unstable, which was related to the colony growth rule. In the process of colony development, the total number of bacteria in the logarithmic growth period is only related to the initial number of bacteria, but after reaching the stable period, the number of bacteria was mainly limited by the colony itself and had little relation to the initial number of bacteria. The sensitive stage of plants to VOCs might be different from the obvious period of bacterial biomass gradient.

Co-culture experiments showed that the VOCs produced by GXUN15128 could promote plant growth stably. VOCs produced by GXUN15128 could promote the growth of *A. thaliana* seedlings in different growth media, but the growth of *A. thaliana* seedlings in the TSA group was better than that in the LBA group. Studies have shown that different growth media affect bacterial VOC function; for example, *Trichoderma aureoviride* exhibits strong inhibitory activity against *Neolentinus lepidus*, *Gloeophyllum trabeum*, and *Coriolus versicolor* in a medium with l-arginine as the nitrogen source, however, the inhibitory activity was weaker in medium with l-phenylalanine as the nitrogen source (65). In our experiment, we found that the difference in seedling growth between the LBA control group and the TSA control group also existed in the absence of inoculation; therefore, the reason may be due to the different VOCs in the culture medium itself. VOCs promoted the growth of *A. thaliana* mainly by increasing stem, leaf, and root length, which has been verified in many studies (34, 52, 66). However, in our study, the gain in root length was not significant or even decreased, but a significant increase was observed in the number of lateral roots. Increases in lateral roots are closely related to the enhancement of plant stress resistance (67–69).

Sixteen compounds were detected by HS-SPME/GC-MS in different media, and the VOC components were different in different media, but 12 of them were identified together. The difference between LBA and TSA medium is the protein source. The protein source of LBA is mostly from animals and yeast, while the protein source of TSA is mainly from plant. The resulting differences in the three VOC components are due to differences in protein sources, further illustrating that amino acid sources can affect microbial volatile secondary metabolites. In this study, different chromatographic peaks identified the same compound, and all of them were pyrazine derivatives. This was because of the identification error caused by their isomer and low signal strength.

Previous studies on VOC identification have mostly used HS-SPME/GC-MS. Briefly, an extraction head coated with activated carbon (ACAR), polydimethylsiloxane (PDMS), and polydivinylbenzene (DVB) was used. The volatile compounds were adsorbed and bound in a VOC environment and then desorbed and released in a high-temperature environment after sampling, separated, and identified. The method can be used to enrich VOCs and can detect the VOC components adequately, but the affinity of adsorbents to different substances is different and cannot represent the true content. Therefore, this was combined with the headspace automatic sampling method to make up the VOC principal-component analysis. In the principal-component analysis of GXUN15128, pyrazine compounds were the main VOCs, which accounted for most of the VOCs. It was concluded that pyrazine compounds are the major VOC components, which promote the growth of *A. thaliana*.

The VOC composition of GXUN15128 was analyzed using the MVOCs database (Table 2) (MVOCs 2.0 database at http://bioinformatics.charite.de/mvoc/) (70). Among the VOCs of GXUN15128, 2-methyl-1-butanol (71), 2-dodecanone (72), 2-nonanol (73), 2,5-dimethyl-3-isopropylpyrazine (74), and 2-methoxy-phenol (75) have been reported in MVOC studies, but they have not been reported as volatiles to promote plant growth. However, it has been reported that benzaldehyde can inhibit mycelium growth of Paecilomyces lilacinus and *Pochonia chlamydosporia* (76) and reduce movement or death of *Panagrelleus redivivus* and *Bursaphelenchus xylophilus* (77). The VOCs of *Azospirillum brasiliensis* also contain 2-ethyl-1-hexanol, and the VOCs released by *Azospirillum brasiliensis* can promote the growth of *Chlorella* and increase chlorophyll A (78).

**TABLE 2:** The VOC composition of GXUN15128 and its research report

VOC	Microorganism^*a*^	Effects^*a*^	Reference
2-Methyl-1-butanol	Bacillus subtilis	NA	71
2-Dodecanone	Serratia marcescens	NA	72
2-Nonanol	Leuconostoc citreum	NA	73
2,5-Dimethyl-3-isopropylpyrazine	Nannocystis exedens	NA	74
2-Methoxy-phenol	Carnobacterium divergens	NA	75
Benzaldehyde	Alcaligenes faecalis	Inhibition of mycelium growth of Paecilomyces lilacinus and *Pochonia chlamydosporia*	76
	Bacillus simplex	Reduction of movement or death of *Panagrelleus redivivus* and *Bursaphelenchus xylophilus*	77
1-Hexanol, 2-ethyl-	Azospirillum brasilense	To promote the growth of *Chlorella* and increase chlorophyll a	78
1-Octen-3-ol	*Tuber borchii*	Inhibit the development of *A. thaliana* and modify its oxidative metabolism	79
1-Nonanol	Pseudomonas sp.	Regulates the jasmonic acid signaling pathway and promotes lateral root formation	33
2-Methyl-3-isopropylpyrazine	NA		
2-Isobutyl-3-methylpyrazine	NA		
2,3,5-Trimethyl-6-propylpyrazine	NA		
2-(2-Methylpropyl)-3-(1-methylethyl) pyrazine	NA		

a In the “Effects” column, “NA” indicates that the substance was identified among this microbe’s VOCs but had no reported function, and in the “Microorganism” column, “NA” indicates that the substance has not been found in MVOCs.

1-Octen-3-ol, found in fungal VOCs, inhibits the growth of *A. thaliana* cotyledons and roots and strongly increases peroxide content in leaves (79). The VOC 1-nonanol produced by Pseudomonas can induce the regulation of the jasmonic acid signaling pathway and promote the formation of lateral roots (33). 2-Methyl-3-isopropylpyrazine, 2-isobutyl-3-methylpyrazine, 2,3,5-trimethyl-6-propylpyrazine, and 2-(2-methylpropyl)-3-(1-methylethyl)-pyrazine have never been reported in microbial VOCs before (Table S3 in the supplemental material). Except pyrazine derivatives, no other compounds have been reported to promote the growth of *A. thaliana*. However, pyrazine and its derivative 2,5-dimethylpyrazine have been reported to inhibit growth of *Burkholderia* and promote potato seedling growth (29, 80). Pyrazine compounds were the main components of VOCs in this study, so they may be the main substances to promote the growth of *A. thaliana*. However, due to the pure substance of the pyrazine derivatives detected in the VOCs of GXUN15128 was not available, it is not possible to perform verification experiments. But through the verification of other compounds except pyrazines, we know that there are no compounds with significant growth-promoting function. Therefore, based on Daly’s research on the plant growth-promoting potential of pyrazines (29), we infer that pyrazines are the main growth-promoting substances in GXUN15128 VOCs.

In this study, the transcriptome of *A. thaliana* seedlings changed after exposure to VOCs. It should be noted that the pathways enriched by DEGs in the KEGG enrichment analysis were all photosynthesis-related pathways, reflecting that VOC-dependent growth promotion of *A. thaliana* is related to photosynthesis.

Photosynthesis antenna protein is the site where plants receive light energy (81), but in this study, *LHCA1*/*LHCA3*/*LHCA4* and *LHCB1*/*LHCB2*/*LHCB3*/*LHCB4*/*LHCB7* expression was downregulated, which may be due to the protective mechanism caused by excessive photosynthesis. Glick (82) found that there was a glut of photosynthesis antenna protein, and that photosynthetic electron transfer could still proceed normally when chlorophyll molecules in PSI and PSII were reduced to one-quarter, respectively. This kind of photosynthesis antenna protein redundancy is beneficial to plants to maintain the competitive advantage of species (83, 84). Ort suggests that plants avoid light damage and energy waste by reducing the volume of chlorophyll-trapping complexes (85) and found that plants could effectively improve the efficiency of the photosystem, the efficiency of photoelectric transmission, and the photosynthetic rate (83, 86–88). Similar to this study, *LHCA2* and *LHCB1*/*LHCB2*/*LHCB4*/*LHCB5*/*LHCB6* were downregulated in rice treated with alternate wetting and drying (AWD) compared with rice treated with continuous flooding (CF) to avoid light damage (89). In the study of the effects of light duration on the transcriptome and proteome of *A. thaliana*, it was also found that the expression levels of *LHCA2*/*LHCA3*/*LHCA4*/*LHCA5* and *LHCB1*/*LHCB2*/*LHCB4*/*LHCB5*/*LHCB6* were lower than those of short or no light after 8 h of illumination (90).

For the circadian rhythm plant pathway, Kim elaborated on the close relationship of the circadian clock with photosynthesis, plant development, and growth metabolism, revealing the direction in which the circadian clock is used to increase crop yield (91). The central oscillator, which consists of *CCA1*, *LHY*, and *TOC1*, is considered to be the core of the circadian rhythm plant pathway. Moreover, other members of the PRR family that TOC belongs to also play a role in plant circadian clocks (92), and deletion of *PRR975* significantly increases tricarboxylic acid (TCA) cycle intermediates and increases chlorophyll, carotenoid, abscisic acid, and tocopherol (93). *CCA1*, *LHY*, and *PRR5*/*PRR7*/*PRR9* collectively control the process of photoperiod, with each *APRR* transcript beginning to accumulate in the order *APRR9*-*APRR7*-*APRR5*-*APRR3*-*APRRR1*/*TOC1* after dawn (94). *CCA1* and *LHY* are positive regulators of *PRR5*/*PRR7*/*PRR9*, while *PRR5*/*PRR7*/*PRR9* are negative regulators of *CCA1* and *LHY* (95). However, in this study, *PRR5*/*PRR7*/*PRR9*, *CCA1*, and *LHY* were all downregulated, which may be due to the knock-on effect of the downregulation of photosynthesis antenna protein gene expression, resulting in the downregulation of *CCA1* and *LHY* expression, which in turn caused *PRR5*/*PRR7*/*PRR9* lack of positive regulation and downregulated expression. Although no relevant studies have confirmed that circadian regulatory genes are negatively regulated by photosynthesis, the reduced red-light sensitivity exhibited by *PRR5*/*PRR7*/*PRR9* deletion mutants illustrates their association (92). The simultaneous downregulation of *PRR5*/*PRR7*/*PRR9* is also consistent with Norihito’s conclusion that these three genes are synergistic (92).

Also, in the circadian rhythm plant pathway, constitutively photomorphogenic 1 (*COP1*) binds UV-B receptor UVR8, thereby promoting the accumulation of *HY5* and *HY5* homologs (*HYH*) (96, 97). *HY5*/*HYH* accumulation, in turn, promotes Gibberellic acid (GA) degradation, stabilizes growth-inhibiting DELLA proteins, and inhibits the function of plant pigment-interacting factors (PIFs), which is not conducive to the growth of plants (98). In this study, downregulation of the expression of *COP1* and *HYH* avoided inhibition of the DELLA protein and inhibited the function of PIFs, thus favoring *A. thaliana* growth.

Chalcone synthase (*CHS*) is the only enzyme whose expression is upregulated. CHS is a key enzyme in the flavonoid metabolic pathway, and *CHS* expression is upregulated by UV/blue light irradiation, different types of light, and pathogen infection (99, 100). Upregulation of *CHS* expression induces the salicylic acid defense pathway and participates in plant disease resistance (101). There is an unknown relationship between *CHS* and the circadian rhythm plant pathway; for example, *CHS* deletion causes upregulation of *CCA1* and *TOC1*, and chlorophyll cycling abolishes the chlorophyll cycle (102). This is similar to upregulation of *CHS* expression and downregulation of *CCA1* expression in this experiment. *CHS* may be a key gene regulating circadian rhythm and photosynthesis.

In the study of antimicrobial activity of Paenibacillus peoriae IBSD35, we know that its antimicrobial substances are antimicrobial peptides, including fricidin, gramicidin, bacteriocin, andracin, fengycin, griselymicins, micrococcin, paenibacterin, ericin, penaeidin, lipopeptide, lassopeptide, plantaricin, and lantibiotics (47). The results of the experiment using Morita’s method of fungistatic function proved (39) that GXUN15128 VOCs had no significant inhibition effect on 10 kinds of pathogenic fungi (Fig. S5). The results showed that the antimicrobial activity of Paenibacillus peoriae was achieved by contact inhibition through secretion of antimicrobial substances but not by volatile secondary metabolites. GXUN15128 shows excellent function in VOCs promoting plant growth and antibacterial activity, so it has great potential to adapt to multienvironmental and agricultural applications.

## MATERIALS AND METHODS

### Isolation and identification of strains.

GXUN15128 was isolated from the rhizosphere soil of *Kandelia candel* in the Tieshan Harbor area, Beihai City, China. About 30 g of soil samples was taken at a depth of 10 to 15 cm, scooped into a sealed bag, and transported back to the laboratory at 4°C for preservation. Two grams of soil samples was added to 20 mL of 0.85% NaCl (wt/vol), kept in a shaker at 30°C for 1 h, and applied as a stock solution. After dilution with sterile distilled water to 10^−2^, 10^−3^, 10^−4^, and 10^−5^, dilutions of different concentrations were spread on TSA medium and cultured at 30°C for 3 days. The growth-promoting function of VOCs from 60 strains was tested by coculture in divided petri plates (I-plate). The growth-promoting strains were screened by the method of phenotypic observation, and the growth-promoting effect was evaluated by the entropy weight method with four parameters, including leaf length, leaf width, root length, and fresh weight, and a strain with a strong plant growth-promoting effect was screened and named GXUN15128. The methodology and evaluation results of the entropy weight method are presented in Table S1 in the supplemental material. In the coculture experiment, 10 μL of 10^8^ CFU/mL bacterial suspension was inoculated on TSA medium, and microbial and plant treatment methods are the same as described later in “Plants and bacteria interact through MVOCs.” Part of the 16S rRNA of GXUN15128 was determined and classified at the species level (1,446 bp; GenBank/EMBL/DDBJ accession number OP210312). To classify GXUN15128 more accurately, we assayed the whole genome of GXUN15128 and calculated average nucleotide identity (ANI) and digital DNA-DNA hybridization (dDDH). The whole-genome of GXUN15128 was sequenced using an Illumina NovaSeq6000 by Majorbio (Shanghai, China), and raw genome-wide data and genome-wide assembly data are deposited in the National Genomics Data Center (NGDC) Sequence Read Archive (https://ngdc.cncb.ac.cn/) and assigned to BioProject accession number PRJCA012772 and BioSample accession number SAMC959306. The strains closely related to GXUN15128 in *Paenibacillus* were selected, and the ANI and dDDH were calculated using Chun Lab’s online ANI calculator (JSpeciesWS, taxonomic thresholds [https://jspecies.ribohost.com/]) (103) and genome-genome distance calculator 3.0 (https://ggdc.dsmz.de) (104). GXUN15128 was observed by scanning electron microscopy (SEM) to determine morphology. Gram staining was also characterized. The media potato dextrose agar (PDA), tryptose soya agar (TSA), Luria-Bertani agar (LBA), and Murashige and Skoog (MS, Hopebio, China) were all used in this study; the agar content was 1.5%.

### Plants and bacteria interact through MVOCs.

The effects of MVOCs on the growth of *A. thaliana* were examined by reference to the seedling and microorganisms coculture experiments in the study by Ryu (105). Artificial climate box parameters were set to a light intensity of 8,000 lx, a temperature of 23°C, and a humidity of 50%, using 16 h of light and 8 h of dark as a cycle. The effects of GXUN15128 VOCs on the growth of *A. thaliana* were analyzed by using 90-mm I-plates. *A. thaliana* seeds were incubated in MS medium for 3 days to form *A. thaliana* seedlings. *A. thaliana* seedlings were transplanted to the MS side (5 plants per plate) and cultured for 2 days under the same condition. Paper discs (8 mm) were placed in the center of one side of the culture medium. The inocula was cultured at 30°C and 200 rpm for 2 days and centrifuged at 5,000 rpm for 5 min; the supernatant was discarded to obtain the bacteria. Sterile water was added, shaken, and mixed, repeating the above steps to obtain pure bacteria. A small amount of sterile water was added to the mixture, and the above steps were repeated. Sterile water was used to dilute bacteria to an optical density at 600 nm (OD_600_) of 0.5 (~10^8^ CFU/mL) as standby (26).

The effects of VOCs from different inocula on plant growth-promoting function were tested by three experimental groups with different inoculations of 10 μL, 7 μL, and 4 μL. Two kinds of microbial media LBA and TSA were used to study the change of VOC composition and the effect of GXUN15128 on promoting plant growth in different media. Sterile water treatment was used as a control, and the experimental method and culture conditions were the same as the experimental group. Each experimental treatment was repeated three times. After 18 days of culture, the growth of bacteria and seedlings was observed, and the growth parameters of *A. thaliana* were determined. The measured plant growth parameters included leaf length, leaf width, root length, and fresh weight. The length and width of leaves were measured by selecting two representative leaves for each seedling. After the medium was removed, the roots were extended on a black background plate, and the lengths of the roots were measured. The agar was crushed with a pressing spoon, and the root system was extracted completely, extended on the background plate, and pressed gently with absorbent paper to further remove the medium and excess water, ensuring root integrity and cleanliness. Because *A. thaliana* seedlings have low weight, the fresh weight was measured by weighing the whole plate of five seedlings after removing the medium.

### Collection of bacterial volatiles.

To collect as many bacterial volatiles as possible, we inoculated bacteria in 20-mL headspace vials (Agilent, US) for sealed culture. To be consistent with the microbial culture conditions in coculture experiments, we used solid media for bacterial culture and VOC enrichment.

A bacterial suspension with an OD_600_ of 0.5 was prepared according to the previous method, and 200 μL of bacterial suspension was added to the solid culture medium plate with 5-mm thickness for coating. LBA and TSA media were used in the experiment. After 3 days of incubation at 30°C, the uniform area of the bacterial colony on the plate was perforated with a perforator 8 mm in diameter. Headspace vials were used as strain cultivation environments, and each sample bottle was filled with 20 bacterial plugs, sealed with a cover, and cultured at 30°C for 5 days. In the blank control group, the operation was the same as that of the experimental group except the solid medium without bacteria.

### Composition analysis by headspace solid-phase microextraction/GC-MS (HS-SPME/GC-MS).

The headspace vials were equilibrated at 30°C for 30 min, and the solid-phase microextraction probe (ACAR/PDMS/DVB, China) was inserted and extracted for 20 min. MVOC composition was analyzed by GC-MS, the solid-phase microextraction probe was inserted into the injection port, and the preset program was run.

The following conditions were used for GC-MS analysis: gas chromatograph (7890B, Agilent), mass spectrometer (5977B, Agilent), chromatographic column (ultrahigh inert gas chromatography column [HP-5MS UI, Agilent], size of 30 m × 0.25 mm × 0.25 μm), carrier gas of 99.999% He, and a chromatographic column flow rate of 1.2 mL/min. The inlet temperature was 250°C, with an initial temperature of 40°C, which was maintained for 2 min, increased to 60°C at a rate of 20°C/min, maintained for 1 min, increased to 140°C at a rate of 20°C/min, increased to 160°C at a rate of 10°C/min, maintained for 2 min, and increased to 250°C at the rate of 30°C/min, without solvent delay; the total running time was 15 min. The temperature of the quadrupole was 150°C, and the temperature of the transmission line was 280°C. The data acquisition and processing system was MassHunter qualitative analysis 10.0.

Automatic identification and integration of chromatographic peaks of ion chromatograms, and automatically retrieve in NIST08 and NIST17. The matching principle is to search for the compounds with a matching rate greater than 80 in two mass spectrometry databases and to determine the compounds according to the order of matching rate.

### Determination of the relative content of MVOCs.

The main components of GXUN15128 and their relationship between the contents were analyzed by GC-MS with headspace sampler (7697A, Agilent). The setting method included an equilibrium temperature of 40°C, sample loop temperature of 50°C, transmission line temperature of 60°C, equilibrium time of 20 min, cycle time of 30 min, pressure equilibrium time of 10 s, injection time of 20 s, and injection volume of 1 mL. The injection needle was driven and cleaned by high-purity He gas for 5 min.

The following GC-MS analysis conditions were used. The initial temperature was 60°C and was maintained for 1 min, increased to 140°C at a rate of 20°C/min, increased to 160°C at a rate of 10°C/min, maintained for 2 min, and increased to 250°C at the rate of 30°C/min; the solvent delay was 3 min, and the total running time was 13 min. The other program settings were the same as the SPME method. The ratio of peak area was used to express the content relationship among the main components.

### Effects of individual volatile compounds on the growth of *A. thaliana*.

Refer to the Blom method for details (30, 106). Based on the VOC components identified by GC-MS, the following components were prepared to validate individual volatile compounds with growth-promoting activity in *A. thaliana*: benzaldehyde (>99.0%), 1-nonanol (≥99.5%), 2-dodecanone (>98.0%), 2-methyl-1-butanol (98%), 2-ethyl-1-hexanol (≥99.5%), 2-nonanol (>98.0%), 1-octen-3-ol (98%), and 2-methoxy-phenol (98%) (Aladdin) using dichloromethane (DCM) mixed with lanolin of the same volume as the solvent (1.6 g of lanolin in 10 mL of DCM). DCM is used to dissolve compounds, and lanolin slowly releases chemicals. Compounds were formulated into experimental samples at concentrations of 1 mg/mL, 100 μg/mL, 10 μg/mL, 1 μg/mL, 100 ng/mL, and 10 ng/mL to analyze dose effects. All experiments were performed using freshly prepared solutions. The solvent without the compound was used as control check (CK). Similar to coculture experiments, I-plates were used to verify the effect of individual volatile compounds on the growth of *A. thaliana*. The two compartments of the I-plates were used to culture *A. thaliana* and to add organic reagents. Culture conditions on the *A. thaliana* side were the same as the method mentioned above. The other side was not filled with any medium, and a paper disc of 8 mm in diameter was placed in the center. Paper discs were pretreated by dropping 10 μL of target solution and drying at 30°C for 3 min. Each concentration was repeated three times, and the paper disc was replaced every 4 days. After adding volatiles for 14 days, the growth parameters, including leaf length, leaf width, root length, and fresh weight, were recorded.

### Plant transcriptome analysis.

The total RNA extraction and purification of *A. thaliana*, cDNA library construction and quality control, and RNA-sequencing method were performed according to Cordovez’s research methods (27). For each treatment, three replicates were used. Each replicate consisted of three plates with five seedlings each to obtain enough biomass. To be consistent with the plants treated by MVOCs, all *A. thaliana* were collected by I-plate after 18 days of exposure under the same conditions. The whole plant was then used as a transcriptome sequencing sample. The AMPure XP system (Beckman Coulter, Beverly, USA), a NanoPhotometer spectrophotometer (Implen, CA, USA), and an Agilent Bioanalyzer 2100 system were used to purify and evaluate the quality of the cDNA libraries. Sequencing libraries were generated using an NEBNext Ultra RNA library prep kit for Illumina (NEB, USA) and were sequenced on the Illumina NovaSeq 6000 platform.

The software trim-galore was used to remove the adapters from the sequence, and low-quality and poly(N) sequences and sequences shorter than the length threshold (the default is 20 bp) were removed (if one sequence was shorter than the threshold, the corresponding two sequences were removed). A quality score of 20 (Q20) ≥ 85% and a Q30 ≥ 80% were qualified. Sequence alignment of the reference genome was performed using HISAT2 v2.0.5 to construct the index of the reference gene and the alignment of clean reads with the reference genome. Read count normalization used fragments per kilobase per million mapped reads (FPKM) to represent gene expression,

$$\text{FPKM}=\frac{\text{cDNA fragments}}{\text{mapped fragment}\times \text{transcript length}}.$$

The Bioconductor package DESeq2 was used for normalization and differential gene expression analyses (107). The average FPKM ratio of the experimental group and the control group was fold change (FC); genes with a $\log_{2}\left|\text{FC}\right|\text{of}\geq \text{1}$ and an adjusted *P* value of <0.05 were defined as differentially expressed genes (DEGs). GO differential enrichment analysis of DEGs and statistical enrichment of DEGs in the KEGG pathway were performed with ClusterProfiler (3.4.4) (108).

### Real-time quantitative PCR.

Real-time PCR was performed using the BlasTaq 2×qPCR master mix G891 (ABM, Canada), which emits fluorescence to cDNA that can then be detected. The gene *Actin* was used as the reference gene. A representative sample of 11 genes was selected for the experiment. On the basis of GO enrichment results, *AIG2* (associated with inducible resistance), *ATPLC* (associated with resistance to adversity), *CYP71B15* and *CYP81D11* (associated with the cytochrome), and *ATPCB* (associated with cell growth and light reaction) were screened as well as *ATCCA1*, *CDF1*, *ATCHS*, *LHY*, *Aprr9*, and *LHB1B2*, which represent the circadian rhythm plant pathway and photosynthesis antenna protein in the KEGG enrichment analysis. Detection and signal collection was performed with the StepOne Plus real-time PCR system (Thermo Fisher, USA), which was enzyme activated at 95°C for 3 min, denatured at 95°C for 15 s, annealed/extended at 60°C for 1 min, and repeated 40 times with 4 replicates per sample. Finally, the relative expression level of each gene was calculated with the Livak (${\text{2}}^{-\Delta \Delta \mathrm{CT}}$) method. After the relative expression was normalized by $\text{log2}(2^{-\Delta \Delta \mathrm{CT}})$, the heat map was made by comparing with the transcriptome gene $\log_{2}\text{(FC)}$ value to verify the difference in gene expression.

### *In vitro* antifungal activity of GXUN15128.

It has been reported that *P. peoriae* can inhibit the growth of plant pathogens. To verify the inhibitory effect of GXUN15128 on the growth of plant-pathogenic bacteria, the antibacterial effect of GXUN15128 against 10 strains of fungal pathogens was determined by the dual culture technique test *in vitro.* The 10 strains of pathogenic fungi used in this study were obtained from the strains stored in Guangxi Key Laboratory for Polysaccharide Materials and Modifications. Because of the different growth rates of different pathogens, the experiments were performed after 7 days of incubation at 25°C. On 90-mm dishes containing 25 mL of PDA medium, two 8-mm paper discs were symmetrically placed on each side 2.5 cm from the center, with the rest of the positions serving as blank controls. The 10-μL suspension of bacteria was inoculated on the paper discs and cultured at 25°C for 3 days. After that, the mycelial plug (8 mm in diameter) was placed in the center for further culture. When the control hyphae grew to a radius of 35 mm, the length of the hyphae in the experimental group was measured, and the bacteriostatic rate was calculated. This experiment included three replicates. The percent inhibition of growth over control was calculated using the formula

$$\text{percent inhibition}=\left[1-\left(\frac{\text{fungal growth in treatment}}{\text{fungal growth in control}}\right)\right]\times 100\%.$$

The VOC inhibitory activity of GXUN15128 was tested by dual plate test. One plate filled with TSA medium was used to culture bacteria, and the other plate filled with PDA was used to culture plant-pathogenic fungi. GXUN15128 at a concentration of 10^8^ CFU/mL was inoculated and coated, and the inoculation method of pathogenic fungi was consistent with the above-mentioned method. GXUN15128 was cultured at 25°C for 3 days, inoculated with fungi, and cultured in combination until the colony diameter of the control group was 70 mm. The colony diameter was measured vertically, and the bacteriostasis rate was calculated.

### Data availability.

Raw RNA-sequencing data are deposited in the National Center for Biotechnology Information (NCBI) Sequence Read Archive (www.ncbi.nlm.nih.gov/sra) and assigned to BioProject accession number PRJNA843851 and BioSample accession numbers SRR19501149 to SRR19501153. Raw genome-wide data and genome-wide assembly data for GXUN15128 are deposited in the National Genomics Data Center (NGDC) Sequence Read Archive (https://ngdc.cncb.ac.cn/) and assigned to BioProject accession number PRJCA012772 and BioSample accession number SAMC959306.
