# Supplementary material for: Identification and Surveys of Promoting Plant Growth VOCs from Biocontrol Bacteria Paenibacillus peoriae GXUN15128
Source: Microbiol Spectr. 2023 Mar 29;11(3):e04346-22. doi: 10.1128/spectrum.04346-22 (PMC10269716; doi:10.1128/spectrum.04346-22)
Supplement: Supplemental file 1 — Supplemental material. Download spectrum.04346-22-s0001.pdf, PDF file, 1.4 MB [file spectrum.04346-22-s0001.pdf]

Supplementary data:

Supporting Information description
Table S1. The entropy method was used to screen the strains with excellent function from the strains with obvious growth promotion.
Table S2. Average nucleotide identity (ANI) and Digital DNA–DNA hybridization (dDDH) for the calculation of 8 strains of *P. peoriae.*
Table S3. The VOC components of GXUN15128 grown on LBA and TSA medium were analyzed by Headspace automatic injection GC-MS
Table S4. Primers used for Real-Time PCR.
Table S5. QC Reads quality results.
Figure S1. The meteorological chromatogram of GXUN15128 VOCs grown on LBA and TSA medium were analyzed by Headspace automatic injection GC-MS and component analysis
Figure S2. The growth-promoting activity of volatile organic compound.
Figure S3. Statistical chart about growth-promoting activity of volatile organic compound.
Figure S4. Correlation analysis of transcriptome samples.
Figure S5. The anti-microbial effect of VOC from GXUN15128.

1. The entropy method was used to screen the strains with excellent function from the strains with obvious growth promotion

Table S1 Evaluation results of entropy weight method

Species	Score	Percentage
GXUN15128	0.950	177.95%
GXUN11	0.652	90.81%
GXUN15103	0.621	81.53%
GXUN15060-2	0.360	5.19%
CK	0.342	0.00%
GXUN10	0.193	-43.56%

The "entropy weight method" was used to evaluate the promoting activity of the strain, and the scores were calculated according to the growth parameters. The calculation is as follows, X_{ij} represents the value of the *Arabidopsis* j parameter evaluation after VOC treatment of strain i (i = 1,2,3, ..., n; J = 1,2,3, ..., m). Y'_{ij} is the Evaluation Index, Y_{ij} is the standard evaluation index, P_{ij} is the proportion of the evaluation index, e_j is the entropy value of the evaluation index, the weight coefficient is d_j, w_j is the weight of the evaluation index, and the final calculated score is expressed as S_i. The "Percentage" represents the ratio between the difference between the treatment group and the control group and the control group, which is used to indicate the difference between the treatment group and the control group.

The calculation is published as follows: $Y'_{ij} = \frac{X_{ij} - \min(X_{ij})}{max(X_{ij}) - \min(X_{ij})}$; $Y_{ij} = Y'_{ij} + 1^{-8}$; $P_{ij} = \frac{x_{ij}}{\sum_{i=1}^{n} x_{ij}}$; $e_j = -k \sum_{i=1}^{n} p_{ij} In(p_{ij}), k = \frac{1}{In(n)}$; $d_j = 1 - e_j$; $w_j = \frac{d_j}{\sum_{j=1}^{m} d_j}$; $S_i = \sum_{j=1}^{m} W_j \times Y_{ij}$; $Percentage = \frac{S_i - S_{CK}}{S_{CK}} \times 100\%$.

2. Average nucleotide identity (ANI) and Digital DNA–DNA hybridization (dDDH) for the calculation of 8 strains of *P. peoriae.*

Strain 1	Strain 2	ANI (%)	dDDH (%)
GXUN15128	*Paenibacillus peoriae ZF390*	96.04	70.60
	Paenibacillus peoriae FSL A5-0030	97.23	79.40
	Paenibacillus peoriae FSL H8-0551	97.08	78.70
	Paenibacillus peoriae FSL J3-0120	97.23	79.60
	Paenibacillus peoriae FSL R7-0131	97.01	78.70
	Paenibacillus peoriae FSL R7-0321	97.13	78.90
	Paenibacillus peoriae HS311	97.12	78.90
	Paenibacillus peoriae IBSD35	97.17	78.70

Category thresholds: ANI: >95%, for (same) species; DDH: >70%, >79%, for (same) species and subspecies.

3. The VOC components of GXUN15128 grown on LBA and TSA medium were analyzed by Headspace automatic injection GC-MS

A

label	Metabolite	Retention Time (min)	Area (%)	CAS no.	Molecular Formula
1	Benzaldehyde	3.79	11.40%	100-52-7	C7 H6 O
2	2-Methyl-3-isopropylpyrazine	4.42	2.25%	15986-81-9	C8 H12 N2
3	2,3,5-Trimethyl-6-propylpyrazine	5.53	80.39%	92233-82-4	C10 H16 N2
4	2-(2-Methylpropyl)-3-(1-methylethyl)pyrazine	6.26	3.43%	1000108-60-3	C11 H18 N2
	Other Ingredients		2.53%		

B

label	Metabolite	Retention Time (min)	Area (%)	CAS no.	Molecular Formula
1	Benzaldehyde	3.8	5.12%	100-52-7	C7 H6 O
2	2-Methyl-3-isopropylpyrazine	4.42	2.31%	15986-81-9	C8 H12 N2
3	2,3,5-Trimethyl-6-propylpyrazine	5.53	87.10%	92233-82-4	C10 H16 N2
4	2-(2-Methylpropyl)-3-(1-methylethyl)pyrazine	6.26	3.21%	1000108-60-3	C11 H18 N2
	Other Ingredients		2.25%		

A. The difference chromatographic peaks in LB group were identified by mass spectrometry, other ingredients represent the unidentified peak.

B. The difference chromatographic peaks chromatographic peaks in TSA group were identified by mass spectrometry. “Other ingredients” represents the small chromatographic peak below the detection limit that can not be identified.

4. Primers used for Real-Time PCR

Table S4 Primers used for Real-Time PCR

Gene ID/name	Primer sequence（5’-3’）
Actin	GGTAACATTGTGCTCAGTGGTGG
	AACGACCTTAATCTTCATGCTGC
CYP71B15	CTCAAAGGAATGATCTCGGA
	TTCTCTCCTTCTTGTCCCC
ATPCB	CTTCATGCATCTTTGTCCG
	ATTAACAAAGCAGTCGTGGAA
CYP81D11	CTATCCGTCAAGATGAGATCCG
	CGTTCTCTGTTCCATCACCGTA
LHY	TACTTGTCTCCTCCATGGCTA
	ATTAGTATCCATAACAGGACCG
APRR9	TTTTGTGTGTTTTAGGCTTT
	CACTGAACAACTTCCGATG
AtCCA1	TTCTCCATTTCCGTAGCTTC
	TATCGTATATGGCTTCCGAGT
AIG2	TCCATGTATTTCTCCTTCCGA
	TTCCATTCCTCGAAATCCC
ATPLC	CAGTAATAGCAGCATCGAAC
	TTCTAAAGTAAGCACAACAGG
CDF1	ACCATGTGACTATCTCCGAA
	GGTAAAACGCAGGATTCCAC
ATCHS	CTACTACTTCCGCATCACC
	TAGCTTAGGGACTTCGACCA
LHB1B2	CCGTGAGCTAGAAGTTATCCACA
	TGCCCAAGTAGTCCAATCCTC

5. Transcriptome data quality control

The reads from the sequencing down machine are filtered to get high quality reads. after data filtering, we do some basic statistics on the data, and the statistics are shown in Table S3. Each base measured will give a corresponding quality value, and this quality value is a measure of sequencing accuracy. Q20 and Q30 indicate the percentage of bases with quality values greater than or equal to 20 or 30. Generally, the minimum Q30>85% is required to measure the quality control data of the downstream data. According to Table S3, the Q30 of the downstream data are higher than 93% and the data are available.

Table S5 QC Reads quality results

Sample	Raw Reads	Clean Reads	Clean Bases	Q20(%)	Q30(%)	GC Content(%)
CK1	41703208	41540062	5.96G	97.97	93.74	45.21
CK2	43488242	43302722	6.22G	97.79	93.39	45.17
CK3	42993388	42824896	6.13G	98.02	93.9	44.84
T1	45664862	45487434	6.48G	98.04	93.96	44.75
T2	46223772	46048720	6.50G	97.85	93.52	44.61
T3	43782794	43621698	6.24G	98.01	93.83	45.37

Raw Reads: the data volume of the original sequence data; Clean Reads: the amount of filtered sequencing data; Clean Bases: the number of sequencing sequences multiplied by the length of sequencing sequences, and converted to g as the unit; Q20 (%): The percentage of bases with pH red value greater than 20 in the total base. Q30 (%): the percentage of bases with pH red value greater than 30 in the total base; GC Content(%): the percentage of the total number of bases G and C in the total number of bases.

6. The meteorological chromatogram of GXUN15128 VOCs grown on LBA and TSA medium were analyzed by Headspace automatic injection GC-MS and component analysis

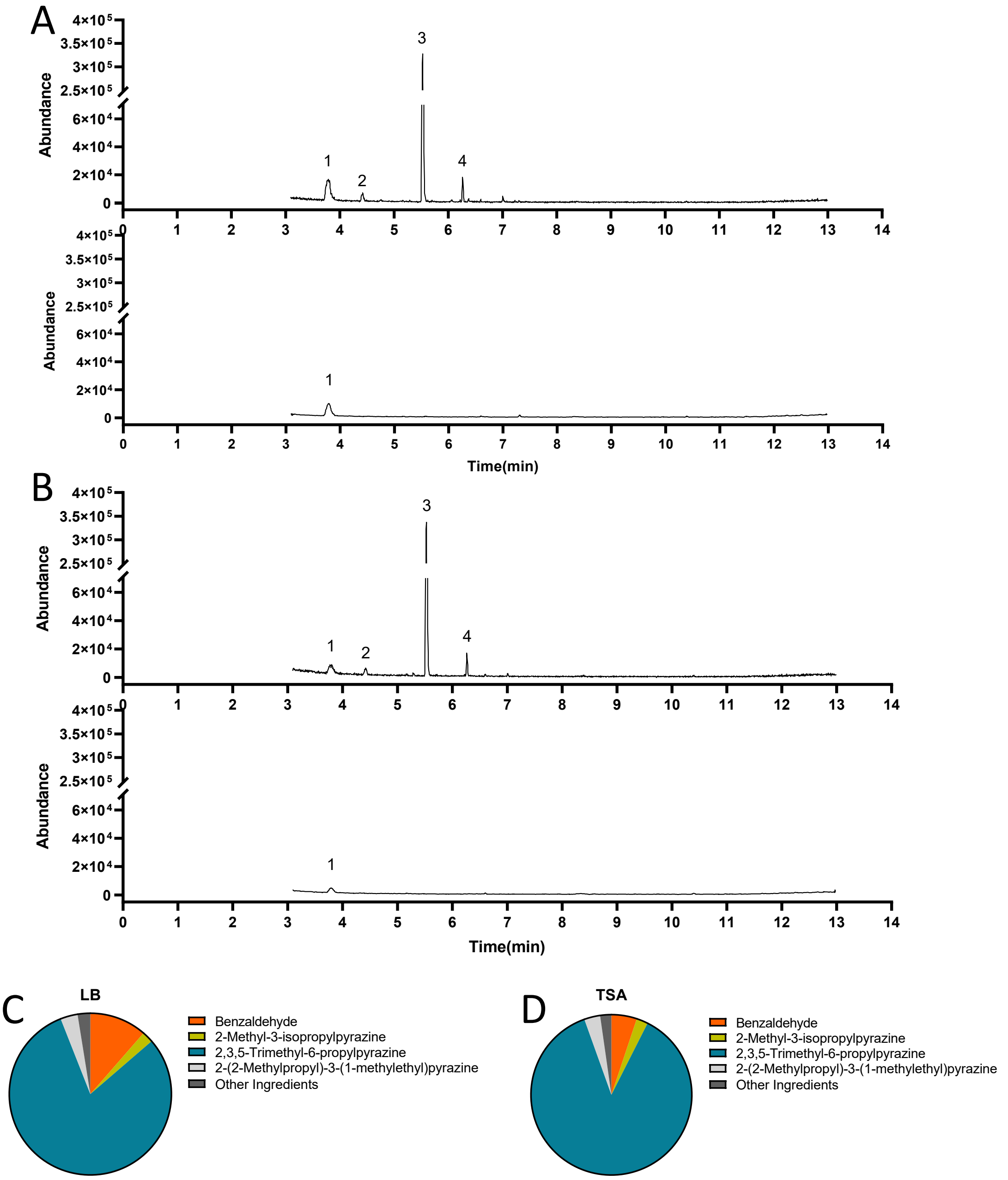

Figure. S1 The meteorological chromatogram of GXUN15128 VOCs grown on LBA and TSA medium were analyzed by Headspace automatic injection GC-MS and component analysis

A. The gas components of GXUN15128 and LB media were detected by Headspace automatic injection GC-MS. The gas chromatograms obtained were compared, the difference peaks were labeled and the components were identified corresponding to (Table S2.A).

B. The gas compositions of GXUN15128 and TSA were detected by Headspace automatic injection GC-MS. The gas chromatograms were compared, and the difference peaks were labeled

and corresponding to (Table S2.B).

C. Pie chart of the relative content relationship of the major components of VOCs produced by GXUN15128 in LB group.

D. Pie chart of the relative content relationship of the major components of VOCs produced by GXUN15128 in TSA group.

7. The growth-promoting activity of volatile organic compound

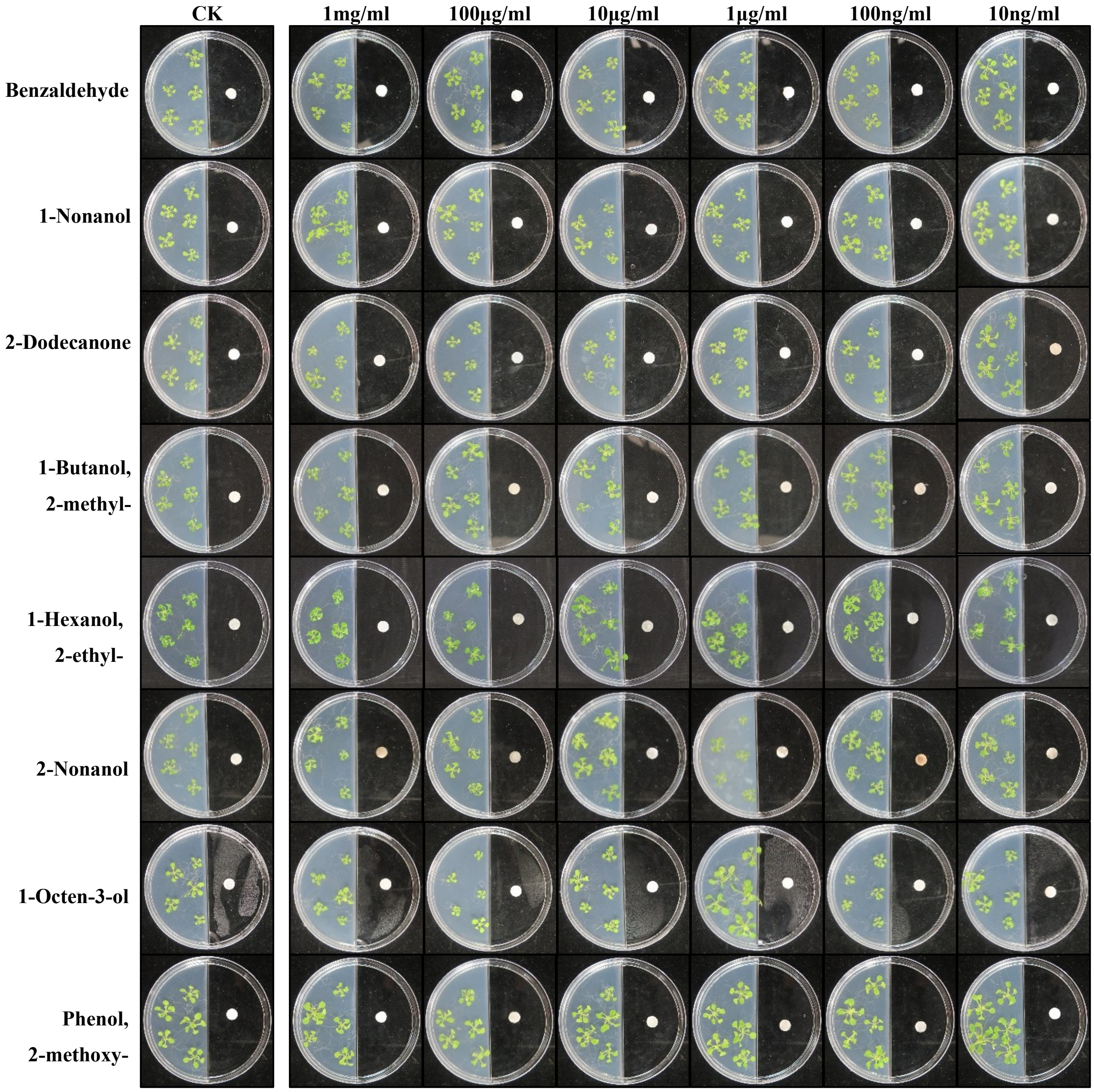

Figure. S2 The growth-promoting activity of volatile organic compound

8. Statistical chart about growth-promoting activity of volatile organic compound

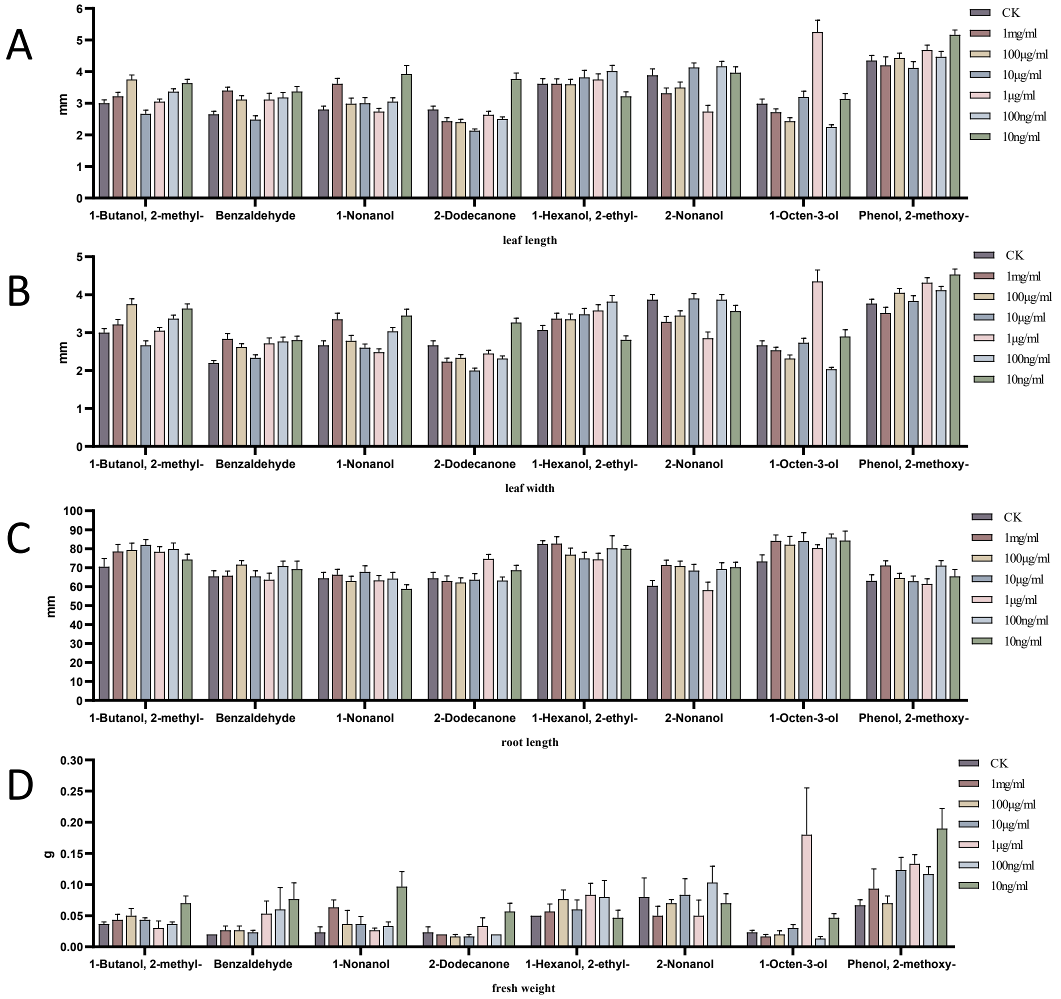

Figure. S3 (A, B, C, D) Statistical diagram of leaf length, leaf width, root length and fresh weight of *Arabidopsis thaliana* treated with pure compounds of GXUN15128 VOCs at concentration gradient.

9. Correlation analysis of transcriptome samples

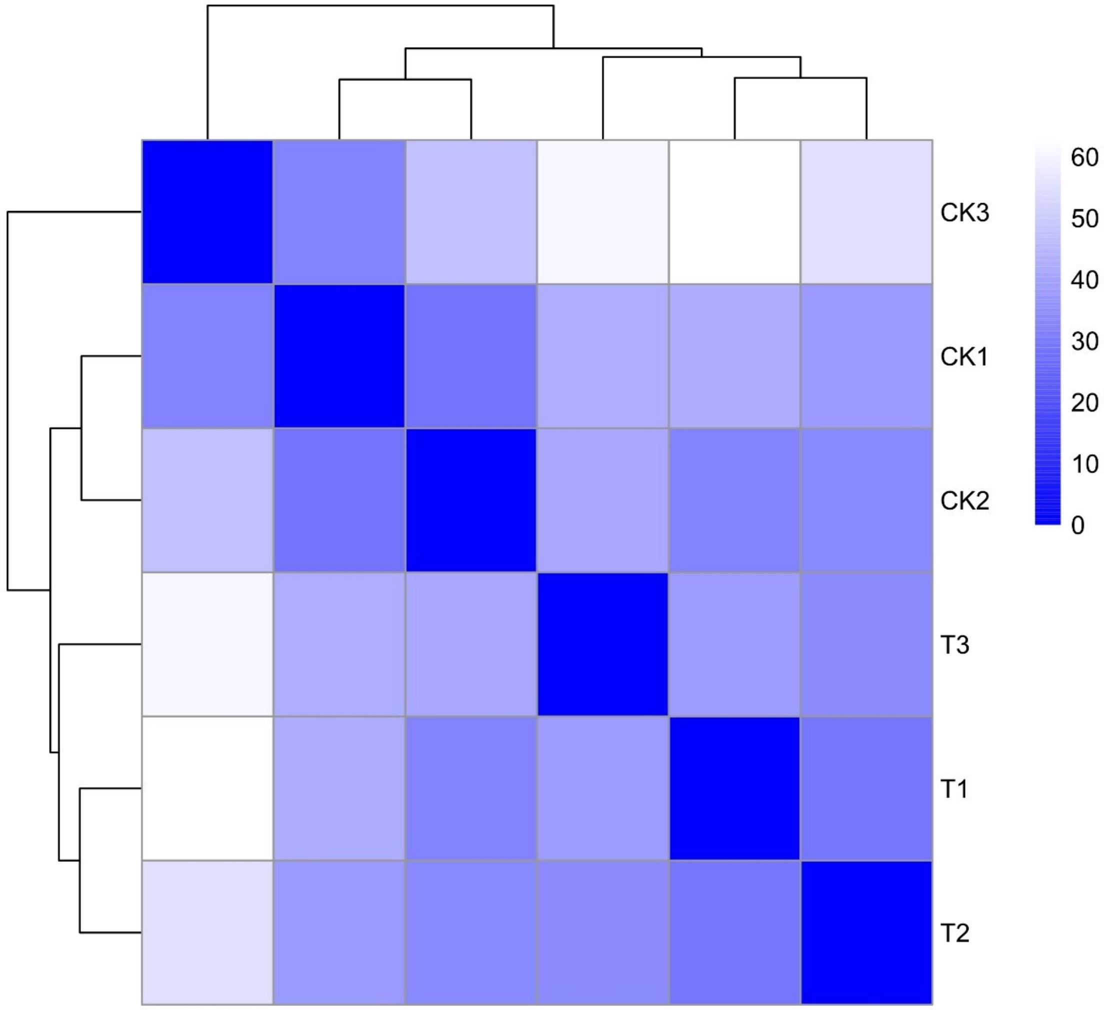

Figure. S4 Heat map of transcriptome differences among *Arabidopsis* samples

10. The anti-microbial effect of VOC from GXUN15128.

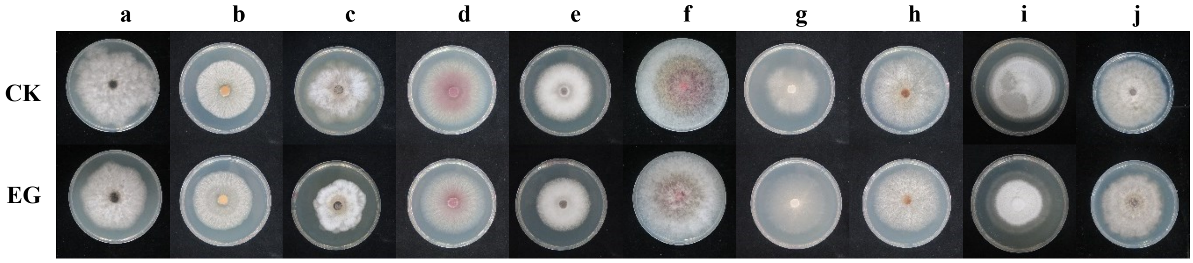

Figure. S5 The inhibitory effect of GXUN15128 Volatile organic compound on the growth of ten pathogenic fungi. “CK” represents control check, “EG” represents experimental group, “a” to “j” respectively for *Cryphonectria parasitica, Neofusicoccum parvum, Botryosphaeria dothidea, Fusarium oxysporum, Alternaria alternate, Fusarium pseudograminearum, Colletotrichum musae, Rhizoctonia solani, Plectosphaerella cucumerina, Bipolaris sorokiniana.*
